# The molecular mechanism of circRHOBTB3 inhibits the proliferation and invasion of epithelial ovarian cancer by serving as the ceRNA of miR-23a-3p

**DOI:** 10.1186/s13048-022-00979-1

**Published:** 2022-06-01

**Authors:** Yihan Fu, Hong Sun

**Affiliations:** 1 Obstetrics and Gynecology Hospital of Fudan University, No. 128 Shenyang Road, Yangpu District, Shanghai, China; 2 Shanghai Key Laboratory of Female Reproductive Endocrine-Related Diseases, Shanghai, China

**Keywords:** Ovarian cancer, Circular RNA, Biomarker, Target therapy

## Abstract

Rising evidences bespeak that circular RNAs are indispensable in regulating cellular biological behaviors and engaging in diseases' occurrence. CircRHOBTB3 has been reported to participate intimately in the progression of some cancers. Nevertheless, the mechanism by which circRHOBTB3 regulates tumorigenesis in epithelial ovarian cancer (EOC) remains ill-defined. The present study determined the expression pattern and bio-effects of circRHOBTB3 in EOC. Furthermore, it revealed that circRHOBTB3 could serve as the ceRNA of miR‑23a-3p to facilitate PTEN expression, suppress proliferation, G1/S transition, invasion, and promote apoptosis in EOC. Summarily, our findings provided a primary research foundation that circRHOBTB3 might be typified as a neoteric biomarker and a promising target of EOC, which is essential for improving the early diagnosis and precision treatment, so as to cut down EOC's mortality finally.

**Supplementary Information:**

The online version contains supplementary material available at 10.1186/s13048-022-00979-1.

## Introduction

Ovarian cancer consists of subtypes with diverse morphological features, clinical-pathological characteristics, molecular specialties, and prognoses [1]. Among these, epithelial ovarian cancer (EOC), especially high-grade serous ovarian cancer (HGSOC), is the most commonly diagnosed type [2]. Despite advances in perioperative and operative techniques, cure rates for EOC have remained flat for more than a decade since most women develop relapse and drug resistance after radical surgery and chemotherapy. Research for addressing this phenomenon has been slow and new methods limited [3]. Thus, we urgently need to further investigate the inner genetic changes that play a vital role in its malignant progression, which will help solve the current dilemma.

Circular RNA (circRNA), regulated by specific cis-acting elements and trans-acting factors, is a type of covalently closed non-coding RNA with cell-specific and tissue-specific expression patterns in eukaryotes. Their dysregulation is closely related to cancers, diabetes mellitus, neurological diseases, cardiovascular disorders ...and so on [4, 5]. Due to its good thermal stability, high expression of encoded antigen, and broad applicability, the circRNA-RBD (Receptor Binding Domain) vaccine also showed its superiority recently. The study done by Qu et al. revealed that the circRNA-RBD vaccine could induce a durable humoral immune response and a solid T-cell immune response in mice [6].

Due to the rapid progress in biotechnology, such as high-throughput sequencing, hybridization, and microarray approaches, emerging circRNAs have been widely identified from animal genomes. Recently, circRHOBTB3, a particularly abundant and evolutionarily conserved molecular, was indicated to be abnormally expressed in prostate cancer (PCa) and clear cell renal cell carcinoma (ccRCC) via the genome-wide circRNA-based microarray analysis [7, 8]. Further analytical measurements like RT-qPCR also successfully validated its prognostic value [8]. Moreover, emerging studies proposed that circRHOBTB3 has been implicated in the evolution of various malignancies. For instance, Deng et al. indicated that circRHOBTB3 exerted suppressive effects on gastric cancer (GC) aggressiveness through stimulating p21 by acting as a sponger of miR-654-3p [9]. Studies revealed that circRHOBTB3 suppressed hepatocellular carcinoma progression by inactivating miR-18a maturation [10]. J. Chen et al. indicated that via HuR/PTBP1 signal pathway, circRHOBTB3 could contribute to the suppression of colorectal cancer (CRC) malignancy [11]. Here, we reviewed the present studies about circRHOBTB3 and presented their conclusions in Table 1.

**Table 1:** circRHOBTB3 in multiple cancer types

Cancer type	up-stream	target	down-stream pathway	effects	Prognosis	Ref
gastric cancer	/	miR-654-3p/p21	/	Proliferation cell cycle	OS AJCC staging,	[12]
pancreatic ductal adenocarcinoma	FUS	miR-600/NACC1	Akt/mTOR	proliferation autophagy	tumor size, vascular invasion, T,	[13]
liver cancer	/	miR-18a	/	proliferation	AJCC staging	[10]
colorectal cancer	/	HuR/PTBP1	/	migration, invasion	staging, T, N, neural invasion, tumor size	[11]
ovarian cancer	/	/	PI3K/AKT	proliferation, migration, invasion		[14]

It has been proposed that circRNAs are actively implicated in innate immunity, cell growth, transformation, and neuronal function by serving as competing endogenous RNAs (ceRNAs) for miRNAs [4]. So we proposed that circRHOBTB3 could also function in such a mechanism. Thus, we went through research about miRNAs, among them, miR-23a-3p attracted our attention. As one of the most widely explored miRNAs, miR-23a-3p was deeply involved in the occurrence and ongoing of various tumors, for instance, hepatocellular carcinoma, renal cell carcinoma, colorectal carcinoma, melanoma cancer, and so on [15–18]. Its abnormal expression in tissues and biofluid may indicate advanced staging, angiogenesis, and metastasis [19]. miR-23a-3p was also implicated in taxol resistance in osteosarcoma and breast cancer [20, 21]. Its abnormal expression was also associated with VPA resistance in epilepsy [22] and TNFα induced insulin resistance in diabetes, [23, 24]. Moreover, a relationship between miR-23a-3p's overexpression with patients’ poor survival and platinum resistance was also observed in ovarian cancer [25, 26]. What’s more, miR-23a-3p’s regulation on PTEN has been detected in various diseases, however, the evidence of this regulation is still weak in EOC. PTEN, one of the most well-known antioncogenes, is lost frequently in malignancies, including EOC [27], PI3K/Akt is a key downstream pathway of PTEN, which has a strong modulation capacity on cell growth, differentiation, proliferation, migration, as well as pro- or anti-inflammatory and metabolism, such as insulin resistance associated with obesity [28].

Nevertheless, the role of circRHOBTB3 and the crosstalk between circRHOBTB3, miR-23a-3p, and PTEN in EOC remains uncertain. To address this question, we obtained 40 epithelial ovarian cancer tissues. According to the FIGO classification, they were divided into stage I ~ II and stage III ~ IV, with 20 patients in each group. Meanwhile, 20 normal ovarian tissues from patients undergoing simultaneous bilateral adnexectomy for cervical cancer were collected as the control group. Then we performed reverse transcription-quantitative PCR (RT-qPCR) to compare the expression level of circRHOBTB3 in these groups. Meanwhile, we obtained their clinical parameters, such as age, lymphatic invasion, distant metastasis, and overall survival time (OS), to explore circRHOBTB3’s correlation with prognosis. Functionally, we knockdown and overexpressed circRHOBTB3 in the EOC cell lines to investigate its effects on proliferation, invasion, cell cycle distribution, and apoptosis in EOC. In-vivo experiments were also carried out to confirm the results. Then we performed the duel-luciferase assay, FISH, and other experiments to verify the directly targeting relationship within circRHOBTB3, miR-23a-3p, and PTEN. Subsequently, we carried on function rescue experiments to disclose the original mechanism that circRHOBTB3 influences EOC’s biological behaviors by serving as the ceRNA of miR-23a-3p,

Collectively, we aimed to indicate that circRHOBTB3 has the promising value to be viewed as a prognostic biomarker and be utilized as a novel therapeutic target in EOC and finally improve patients’ clinical outcomes in the future.

## Material and methods

### Tissues and cell culture

A total of 60 human ovarian tissue samples were obtained from patients who had surgery in the Obstetrics and Gynecology Hospital of Fudan University. Patients’ clinical data was obtained afterward. The classification of clinical staging and histological grading of ovarian cancer were determined according to the FIGO 2014 system. All excised specimens were immediately frozen in liquid nitrogen and then stored at − 80 °C until use.

Human ovarian cancer cell lines (A2780, OVCAR8, SKOV3, SKOV3-ip) and human normal ovarian cell line IOSE-80 were obtained from American Type Culture Collection (ATCC). The medium was a mixture of 10% fetal bovine serum (FBS) (Gibco, Life Technologies, USA), 1% penicillin, 1% streptomycin, 1% amphotericin B, and RPMI 1640 medium. Cells were cultured at 37 °C with 5% carbon dioxide. 

### Reverse transcription-quantitative PCR

First, total RNA was extracted by RNA Purification Kit (EZBioscience, USA). Then, after RNA isolation, a total RNA of 10 μg was incubated for 45 min at 37ºC with 2 U/μg RNase R (Epicentre Technologies, RNR07250). cDNA synthesis was implemented using PrimeScript RT Reagent Kit (RR037, Takara, Dalian, China). For microRNA, reverse transcription was carried on by using miRNA 1st Strand cDNA Synthesis Kit (Vazyme, Nanjing, China). Quantitative RT-PCR was conducted using Taq Pro Universal SYBR qPCR Master Mix (Vazyme, Nanjing, China) with a 3-step cycling protocol. GAPDH was used as an internal reference for circRNA and mRNA expression, U6 was used as an internal reference for miRNA expression. Primers used in this study are as follows:

- GAPDH-S: GGAAGCTTGTCATCAATGGAAATC;
- GAPDH-A: TGATGACCCTTTTGGCTCCC;
- RHOBTB3-S: TGAACTCCACAGCCTTGATGAC:
- RHOBTB3-A: GGCAGCAGAACAGCAAGTTATTT;
- hsa_circ_0007444-S: ATTCAGGTGCTTTTCAGTGGG;
- hsa_circ_0007444-A: GGCAGCAGAACAGCAAGTTATTT;
- U6-S: CTCGCTTCGGCAGCACA;
- U6-A: AACGCTTCACGAATTTGCGT
- hsa-miR-23a-3p-RT: CTCAACTGGTGTCGTGGAGTCGGCAATTCAGTTGAGGGAAATCC;
- hsa-miR-23a-3p-S:
- ACACTCCAGCTGGGATCACATTGCCAGGG
- PTEN-S: AGGGACGAACTGGTGTAATGA
- PTEN-A: CTGGTCCTTACTTCCCCATAGAA

### RNA Fluorescence in situ Hybridization (FISH)

Paraffin sections need to be dewaxed and digested first, while the cell slides need to be fixed with 4% paraformaldehyde. A fluorescence in situ hybridization kit (Servicebio, Wuhan, China) was used for following procedures. First, the pre-hybridization solution was added and incubated at 37 °C for 1 h. After removal, the hsa-circ-0007444 probe hybridization solution with a concentration of 1.5uM was added and hybridized overnight at 42 °C. After washing, the miR-23a-3p probe hybridization solution with a concentration of 1.5uM was added and hybridized overnight at 42 °C. After washing using SSC, the section was incubated with DAPI for 8 min in the dark, then mounted. Finally, images were taken with the confocal microscope (Nikon Instruments, Japan). At least five different visual fields were taken for each slice.

The FAM-labelled circRHOBTB3 probe and cy3-labeled miR-23a-3p probe were designed and synthesized by Servicebio (Wuhan, China). The probe sequences are listed below:

- hsa_circ_0007444: 5’-AGGCATTTTTTCTTTCCTGGTGTTTT-3’;
- miR-23a-3p: 5’-GGAAATCCCTGGCAATGTGAT-3’.

### Transfection

Before transfection, cells were inoculated in the culture medium free of antibiotics overnight. The cell density should be about 70% on the day of transfection. During transfection, first, plasmid or siRNA was diluted with Opti-MEM medium and stood for 5 min. Lipofectamine 2000 (Invitrogen, CA, USA) was also diluted with Opti-MEM medium and left for 5 min. The transfection complex could be obtained by mixing and incubating these two solutions at room temperature for 20 min. Then, the transfection complex was evenly added to the cell culture medium. 6–8 h later, changed the fluid. And cells could be collected for functional experiments after 48 h.

Plasmids, miRNA mimics, and siRNA used in this study were synthesized by PPL (Public Protein/ plasmid Library, China). Related information like sequences and vectors is provided in supplementary file 1.

### Stable transfection

The SKOV3 cells in the logarithmic growth phase and in good condition were laid to a 24-well plate, and its density should reach 50% on the second day.

On the second day, the old medium was displaced with 0.5 ml fresh culture medium containing Polybrene (3 µg/ml) per well. And the medium containing the lentivirus was prepared according to the multiplicity of infection (MOI) value. First, 250 µl/well medium was added and incubated for 4 h. After 4 h, another 250 µl medium was added. After 24 h, changed the fluid. After 48 h, 2.5ug/ mL Puromycin was added in the medium for screening. After 3 generations of culture, screening was stopped, and stable strains were harvested for verification.

PPL (Public Protein/Plasmid Library, China) assisted lentivirus packaging, concentration, and titer determination.

### Dual-luciferase reporter assay

Initially, the pmirGLO luciferase reporter plasmid, which contained the potential circRHOBTB3 binding site with miR-23a-3p, was constructed (HanBio, Wuhan, China). 293 T cells were incubated in 96-well plates and then co-transfected the wild-type (wt) or mutant-type (mt) circRHOBTB3 reporter plasmid with miR-23a-3p mimic or mimic NC. Luciferase detection was performed using Luciferase Assay Reagent (Promega, WI, USA). After 48 h of culturing, Firefly/ Renilla luciferase activities were recorded according to the manufacturer’s instructions. The data are shown as mean ± SEM of at least three independent experiments. Related information like sequences and vectors is provided in supplementary file 2.

### CCK8 assay

Cell viability was measured by the CCK-8 assay kit (Dojindo, Japan). 6 h after transfection, the adherent cells were digested and adjusted to 20000 cells/ml, then 100μl/well suspension was added and incubated in the 96-well plate. At harvest time (every 24 h after incubation), the ordinary culture medium was removed. Later, 10 µl CCK-8 solution dissolved in 100μl medium was added per well, then the plate was incubated at 37 °C for 30 min. Afterward,  the absorbance was measured at 450 nm, and cell viability was calculated. The data are shown as mean ± SEM of at least three independent experiments.

### Matrigel invasion assays

Cells were starved for 6 h before resuspending, and 50 µl of Matrigel (BD, USA) was pre-applied to the upper chambers. After gelatinous curing, a total of 1 × 10^5^cells/well in 200 ul of serum-free medium were seeded into the upper Transwell chamber (Corning, USA). Meantime, 600 ul medium with 20% FBS was added to the lower chamber of the 24-well plate. After 48 h, cells on the upper surface were gently wiped, while cells on the lower surface were fixed with 4% paraformaldehyde for 20 min and then stained with 0.1% crystal violet for 20 min. Finally, an inverted microscope (Nikon, Japan) was used to photograph five random areas for each chamber. The data are shown as mean ± SEM of at least three independent experiments.

### Flow cytometry

Cell cycle distribution was detected using a cell cycle analysis kit (Solarbio, China). OC cells were fixed in ice-cold 70% anhydrous ethanol, then stored at 4℃ before DNA was stained with PI/RNase staining solution at 37℃ for 30 min away from light. Flow cytometry (BD Biosciences, San Jose, CA, USA) was utilized to obtain data. The data are shown as the mean ± SEM of at least three independent experiments.

### Apoptosis

Apoptosis was measured using Annexin V-EGFP kit (EpiZyme, China).

The cell culture medium was collected in the centrifuge tube before the cells were digested. The supernatant was discarded after suspension with the original culture medium, then the cells were collected by centrifugation after washing with PBS for 2 times. Annexin V-EGFP and Propyl iodide (PI) was added into 192 μL binding Buffer and thoroughly mixed to obtain the Staining Buffer. 200 μL Staining Buffer was added to 0.5 × 10^5^–1 × 10^5^ cells. The cells were incubated at room temperature for 5–10 min and then detected by flow cytometry immediately.

### Western blot assay

After total protein was isolated using Radio-Immunoprecipitation assay (RIPA) buffer (Beyotime, China). Then, 30 μg of protein were segregated by 10% SDS/PAGE gels and transferred to PVDF membranes (Millipore, USA), blocked with TBST buffer containing 5% skim milk powder incubated with primary antibodies against PTEN (1:750 dilution), AKT (1:800 dilution); p-AKT (1:800dilution); GAPDH (1:40,000 dilution) at 4 °C overnight with a primary antibody. After washing, the membrane was hybridized with HRP-conjugated secondary antibodies (1:3000 dilution) for 30 min at 37 °C. The signals were visualized using the Western Blotting Detection Kit (Solarbio, Beijing, China). The relative protein expression levels were normalized to the GAPDH level. The experiment was repeated three times.

### In vivo experiment

10 BALB/c nude mice were divided into two groups, each injected with 2 × 10^7^ cells/200 μl of circRHOBTB3 overexpression (OE) or negative control (NC) stable transgenic cells in the abdominal cavity. Subcutaneous tumor formation in nude mice was observed after 3 days, and tumor volume and weight were measured every 3 days after that. Volume measurements: use cursor calipers to measure the longest (a) and shortest (b) diameter of subcutaneous tumors and tumor volume: v = a * b * b / 2. At the end of the experiment, the nude mice were weighed, the nude mice were sacrificed by cervical dislocation, the subcutaneous tumor was carefully peeled off and weighed and immediately immersed in 4% polyformaldehyde preservation.

### Bioinformatic prediction

Starbase: https://starbase.sysu.edu.cn/starbase2/index.php

miRBD: http://mirdb.org

ENCORI: https://rna.sysu.edu.cn/encori/index.php

### Statistical analysis

SPSS 16.0 software was used for statistical analysis. The experimental data were presented as mean ± standard deviation. T-test was used to compare means between two groups, and the one-way ANOVA test compared more than two groups. Quantified values were the mean SD of at least three independent experiments. *P < 0.05, **P < 0.01, ***P < 0.001, ****P < 0.0001 indicated that there was a statistically significant difference.

## Results

### circRHOBTB3 is relatively low-expressed in EOC and correlated with tumor stage

**Fig. 1:**
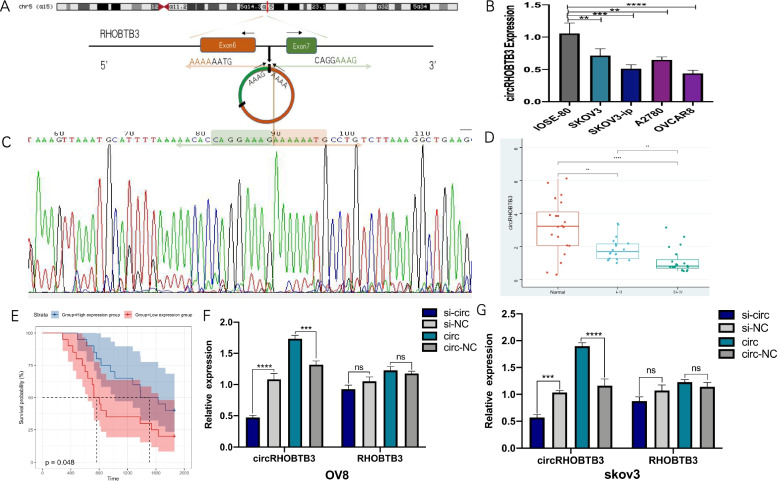
CircRHOBTB3 is down-regulated in EOC. **A** Schematic illustration showing the circularization of RHOBTB3 exon 6 and exon 7 forming circRHOBTB3. **B** Relative expression of circRHOBTB3 in OC cell lines compared with IOSE-80 was measured by qRT-PCR. **C** The existence of circRHOBTB3 was validated by RT–PCR followed by Sanger sequencing. The green and orange box represents “head-to-tail” splicing sites of circRHOBTB3. **D** The expression of circRHOBTB3 was detected by real-time PCR in 40 EOCs and 20 normal ovarian tissues. (one-way ANOVA). **E**. Survival curve depicted the association between circRHOBTB3 with patients’ overall survival time. **F/G**. Relative circRHOBTB3 and RHOBTB3 mRNA expression were detected after transfection in SKOV3 and OVCAR8 cells by qRT-PCR (Student’s t-test). Quantified values were the mean SD of at least three independent experiments. (*P < 0.05, **P < 0.0, ***P < 0.001, ****P < 0.0001)

According to CircBase, circRHOBTB3 was produced from exon 6 and exon 7 of the RHOBTB3 gene (Fig. 1A). Interestingly, we explored in  the MiOncoCirc, the first comprehensive database to analyze circRNAs expression in more than 2000 cancer samples using exome capture RNA sequencing technology, and found out that circRHOBTB3 was relatively low expressed in ovarian cancer (supplementary file 3) [29]. Previous circRNA-seq results also suggested that the low-expression of circRHOBTB3 might be a significant warning of EOC occurrence [30, 31]. Given these, firstly, we detected circRHOBTB3 expression in some common EOC cell lines: SKOV3, SKOV3-ip, A2780, and OVCAR8 (OV8), and compared them with the normal ovarian epithelial cell line IOSE-80 (Fig. 1B). Results showed that the expression of circRHOBTB3 in EOC cell lines was relatively lower than in IOSE-80. We further confirmed the existence of head–tail splicing sequences of circRHOBTB3's RT-PCR products by Sanger sequencing (Fig. 1C).

To furtherly determine whether circRHOBTB3 can be regarded as a candidate circRNA that may be involved in EOC's oncogenesis, we collected 40 EOC tissues, including 20 cases of stage I~II and 20 cases of stage III~IV. In addition, we used 20 normal ovarian tissues from patients who underwent bilateral adnexectomy for cervical cancer as the control group. RT-PCR was conducted to detect the expression of circRHOBTB3 in these 60 ovarian tissues, and results showed that the differences of circRHOBTB3 expression among these three groups were statistically significant (Fig. 1D).

Then, according to the median value of circRHOBTB3 expression in 40 cases of EOC, we divided the patients into a high expression group (*n* = 20) and a low expression group (*n* = 20). Then, Fisher’s exact test was utilized to inspect the correlation between circRHOBTB3 content and various clinical parameters, such as age, lymph node metastasis and distant metastasis. It revealed that low expression of circRHOBTB3 was significantly associated with lymphatic metastasis, and age, but not distant metastasis (Table 2). We subsequently performed survival analysis, the promising value of circRHOBTB3 as a prognosis biomarker was proved (*P* < 0.05) (Fig. 1E). These results prompted us to deepen our exploration of the role of circRHOBTB3 in the progression of EOC.

**Table 2:** Association between circRHOBTB3 expression with multiple clinical parameters (Fisher’s exact test, *P < 0.05, **P < 0.0, ***P < 0.001, ****P < 0.0001)

Parameters	circRHOBTB3		P Value	significance
	Low expression group (*n* = 20)	High expression group (*n* = 20)		
Age				*
≤ 55	9	16	0.0484	
> 55	11	4		
Node invasion				*
N0	9	17	0.0187	
N1	11	3		
Metastasis				/
M0	12	18	0.0648	
M1	8	2		

### The biological effect of circRHOBTB3 on OC cells

**Fig. 2:**
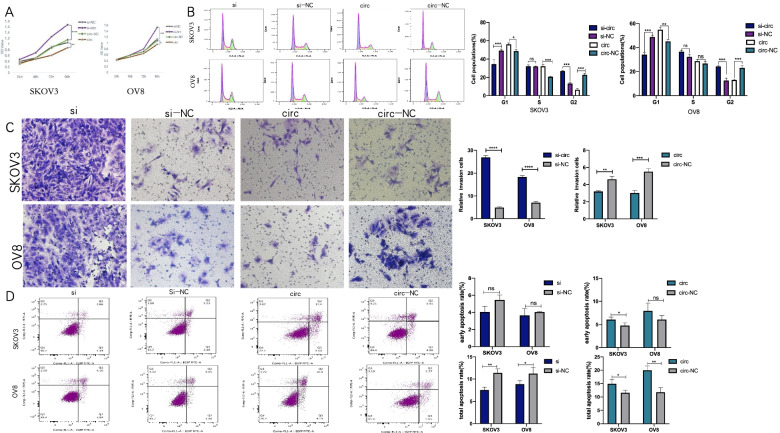
Effects of circHRHOBTB3 on proliferation, invasion, cell cycle distribution, and apoptosis in OC cells. The transfection groups were respectively: si-circ, si-NC, circ, circ-NC groups. **A** The growth curves of cells were analyzed using CCK8 assay (Student’s t-test). **B** The cell cycle distribution was examined by flow cytometry (Student’s t-test). **C** Invasion abilities of EOC cells were evaluated by transwell matrigel invasion assay. (Student’s t-test), **D**. Cell apoptosis was examined by Flow cytometry. Quadrant 1 (Q1) represented a flowchart of dead cells; Quadrant 2 (Q2) represented inactive apoptotic cells; Quadrant 3 (Q3) represented viable apoptotic cells; Quadrant 4 (Q4) represented viable non-apoptotic cells. (Student’s t-test)

Transfection was executed in SKOV3 and OVCAR8 to address the effects circRHOBTB3 exert in EOC’s biological behaviors since they ranked the highest and lowest among the cell lines we exanimated above. The expression of circRHOBTB3 of EOC cells transfected with si-circRHOBTB3 and circRHOBTB3 overexpressing (OE) plasmids was significantly inhibited and up-regulated, respectively, according to the RT-PCR results. Meanwhile, the expression of RHOBTB3 mRNA had no apparent change (Fig. 1F-G). Afterward, the effects of circRHOBTB3 on the proliferation, invasiveness, cell cycle distribution, apoptosis of EOC cells were investigated by functional assays. First, CCK8 assays were carried out to reveal the effects of circRHOBTB3 on proliferation. It showed that the cell proliferation ability was significantly enhanced in SKOV3 and OVCAR8 with circRHOBTB3 knockdown, while overexpression of it caused the opposite effect (Fig. 2A).

Subsequently, cell cycle distribution was measured by flow cytometry. As presented in Fig. 2, fewer cells were engaged in the G1 phase after downregulation of circRHOBTB3 in SKOV3 and OVCAR8 cell lines, which proposed the knockdown of circRHOBTB3 promoted G1/S cell cycle succession. Nevertheless, overexpression of circRHOBTB3 arrested more cells in the G1 phase (Fig. 2B).

Meanwhile, the matrigel transwell assay suggested that the invasive capacity of SKOV3 and OVCAR8 declined prominently with upregulation of circRHOBTB3. In contrast, silence of circRHOBTB3 fostered invasiveness in OVCAR8 and SKOV3 cells (Fig. 2C). 

Finally, we investigated the apoptosis among these groups. Although the early apoptosis rate results were a bit unstable, the total apoptosis rate verified that overexpression of circRHOBTB3 could significantly facilitate apoptosis of EOC cells, while downregulation of it could inhibit apoptosis significantly. Overall, these results demonstrated that circRHOBTB3 was a suppressive regulator for EOC's malignant progression.

### CircRHOBTB3 serves as the sponge of miR-23a-3p

Given that circRNAs mainly exert their biological effects via spongy binding of miRNAs (Li et al., 2015; Xin et al.,2017), we thus proposed that circRHOBTB3 could also function in that way. MiR-23a-3p overexpression was associated with poor survival and platinum resistance in OC patients. More interestingly, its inhibitory regulation on PTEN has been contributed to multiple biological behaviors in various diseases [32–35]. However, the pathway of miR-23a-3p/PTEN in EOC has not yet been well illustrated. Therefore, we came up with that miR-23a-3p/PTEN must be worth exploring as the downstream pathway for circRHOBTB3 in EOC.

**Fig. 3:**
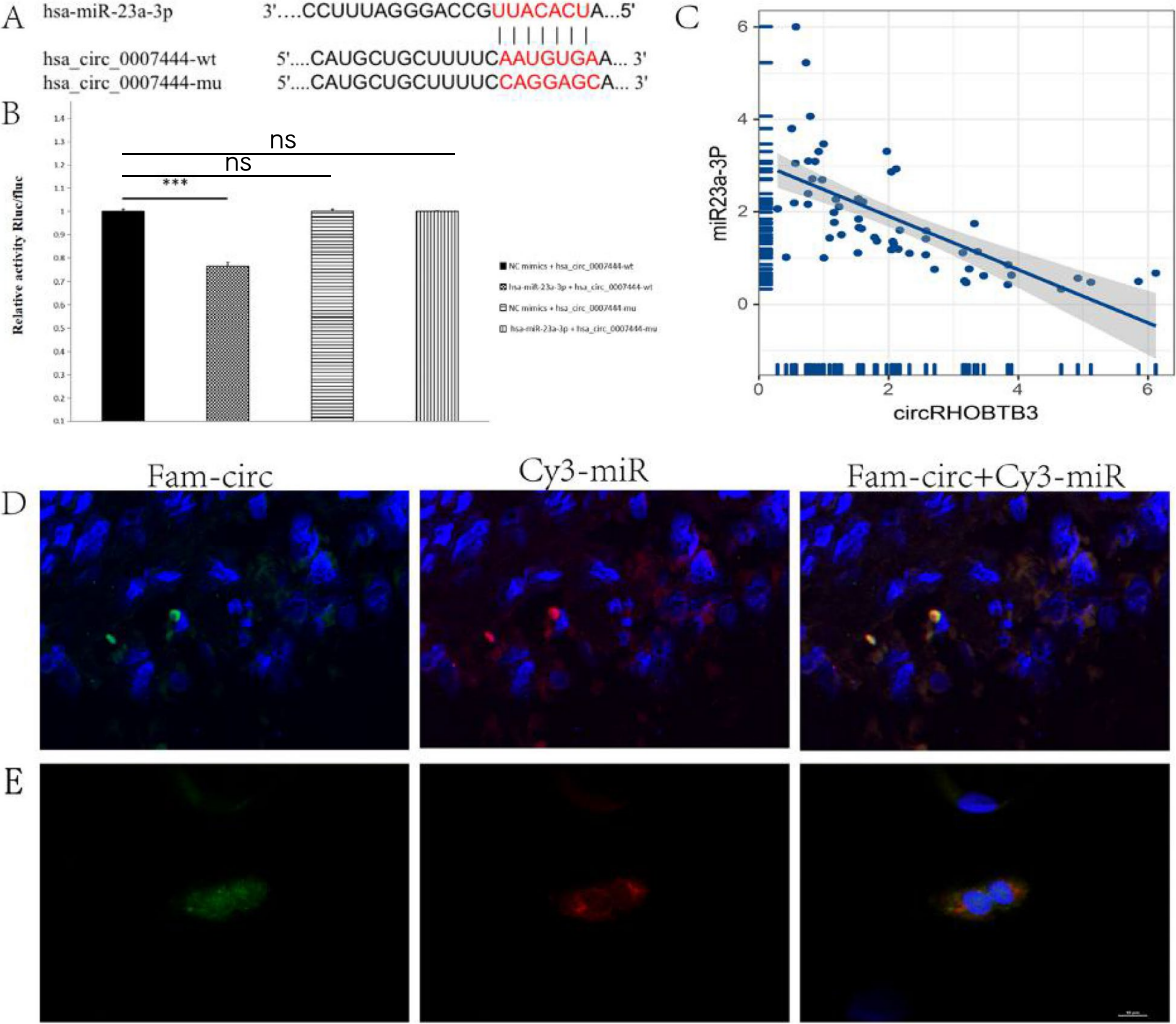
miR-23a-3p is directly targeted by circRHOBTB3. **A** The binding sites of circRHOBTB3 in miR-23a-3p were depicted. **B** The relative luciferase activity was assessed by dual-luciferase report assay. (one-way ANOVA). **C** the relationship between miR-23a and circRHOBTB3 expression in 60 ovarian tissues was analyzed. (Pearson’s correlation analysis). **D** Fluorescence in situ hybridization (FISH) assay performed in paraffin shows the expression of circRHOBTB3 and miR-23a-3p in EOC. CircRHOBTB3 probe was labeled with FAM, which was the green light. MiR-23a-3p probe was labeled with cy3, which was the red light. The nuclear staining by DAPI was blue under ultraviolet excitation, **E** Fluorescence in situ hybridization (FISH) assay performed in cell-climbing section shows the co-localization between circRHOBTB3 and miR-23a-3p

Firstly, the putative binding sites of miR-23a-3p in circRHOBTB3 were forecasted using the StarBase database. Then, we confirmed this binding site using ENCORI and miRBD databases (Fig. 3A). Based on it, we further performed a dual-luciferase reporter assay in which RLu-circRHOBTB3-wt or RLuc-circRHOBTB3-mt plasmid was co-transfected with miR-23a-3p mimic or NC mimic into HEK-293 T cells. As a result, co-transfection with miR-23a-3p mimic markedly reduced the luciferase activity of the circRHOBTB3-wild type but did not affect the mutant (Fig. 3B). Moreover, an inverse expression relationship between circRHOBTB3 and miR-23a-3p was detected in EOC tissues (*r* = -0.58, *p* = 3.767647e-09) (Fig. 3C).

We subsequently performed the FISH assay in EOC’s Paraffin sections, which illustrated the expression and location of circRHOBTB3 and miR-23a-3p in EOC’s cytoplasm (Fig. 3D, supplementary file 4). Cell climbing-FISH assay further demonstrated the co-localization of circRHOBTB3 and miR-23a-3p clearly (Fig. 3E).

Together, these results collectively verified that circRHOBTB3 could directly bind with miR-23a-3p. In the following sections, we will investigate in-depth about the mechanism of circRHOBTB3, serving as the sponge of miR-23a-3p, to regulate EOC’s biological behaviors.

### CircRHOBTB3 inhibited cell proliferation, G1/S transition, and invasion while promoting apoptosis by sponging miR-23a-3p in EOC.

**Fig. 4:**
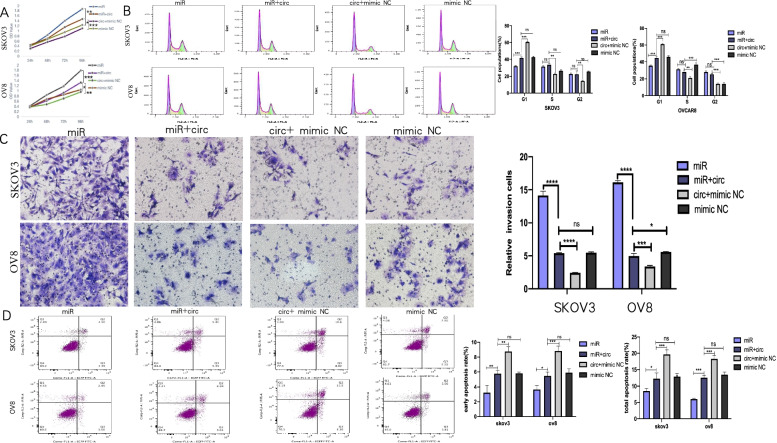
Over-expression of circRHOBTB3 reverses miR-23a-3p-fostered malignancy in OC cells. **A** In the CCK8 assay, proliferation circRHOBTB3 competively reduced the proliferation of SKOV3 and OVCAR8 cells that miR-23a-3p increased (one-way ANOVA). **B** CircRHOBTB3 competitively stagnated cells in the G1 period that could be released by miR-23a-3p (one-way ANOVA). **C** Transwell matrigel invasion assay demonstrated that miR-23a-3p fostered the invasion ability of SKOV3 and OVCAR8 cells; however, when co-transfected with circRHOBTB3, this effect was significantly attenuated (one-way ANOVA). **D** Apoptosis assay was performed to show whether circRHOBTB3 could rescue miR-23a-3p’s inhibition of apoptosis (one-way ANOVA)

Subsequently, we performed the CCK-8 assay to have a penetrating insight on the rescue effect of circRHOBTB3 on proliferation. The OD value trend of SKOV3 and OVCAR8 treated for 24 h,48 h,72 h, and 96 h showed that circRHOBTB3 could effectively reverse the significant elevation in proliferation caused by miR‐23a-3p (Fig. 4A).

Consistently, transwell assay results indicated that the EOC cells co-transfected with miR-23a-3p mimic and circRHOBTB3 OE plasmid showed a decline in invasion capability compared with miR-23a-3p mimics solitarily transfected cells (Fig. 4B), which supported that circRHOBTB3 could effectively rescue the miR-23a-3p promoted invasiveness of OC.

Next, we carried out the flow cytometry assay to determine whether circRHOBTB3 could attenuate miR-23a-3p related G1/S transition speed up. The proportion of OC cells arrested in the G1 phase in the group co-transfected with miR-23a-3p mimic and circRHOBTB3 OE plasmid was between the group transfected with miR-23a-3p mimic and the group transfected the circRHOBTB3 OE plasmid solely, which successfully validated our presumption (Fig. 4C).

Finally, we explored circRHOBTB3's ability in rescuing apoptosis. The results demonstrated that circRHOBTB3 could competitively reverse miR-23a-3p related suppression of apoptosis in EOC (Fig. 4D).

Collectively, it was proved that circRHOBTB3 could affect EOC's proliferation, invasion, cell cycle distribution, as well as apoptosis by competitively sponging miR-23a-3p.

### circRHOBTB3 regulated PTEN/Akt pathway by serving as the ceRNA of miR-23a-3p

**Fig. 5:**
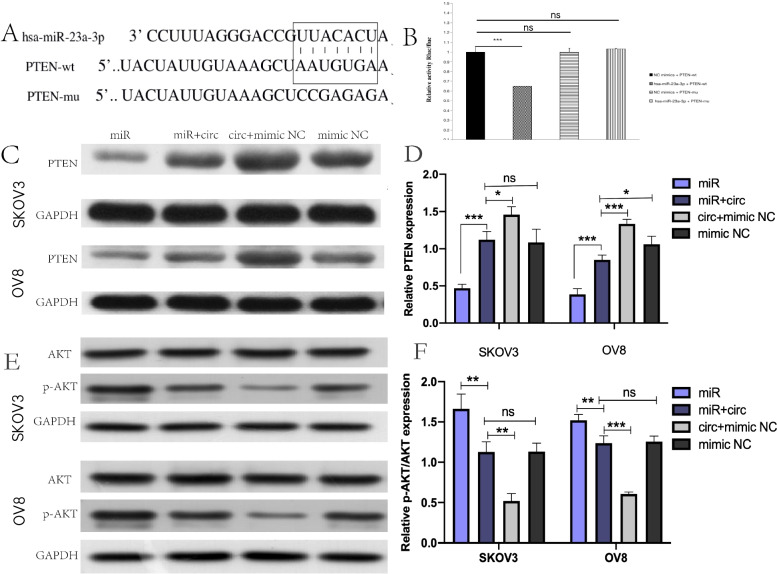
CircRHOBTB3 reversed miR-23a-3p-induced inhibition of PTEN expression in OC cells. **A** The binding sites of PTEN in miR-23a-3p were depicted. **B** Luciferase reporter assay showed miR-23a-3p mimics suppressed the activity of PTEN 3’UTR-WT. However, there was no statistical effect on luciferase activity when PTEN 3’UTR-MT and miR-23a-3p mimics were co-transfected. (one-way ANOVA). **C, D** Relative PTEN expression was detected after transfection by WB (one-way ANOVA). **E, F** Expression of Akt, p-Akt protein was measured in groups by Western blot. in OC cells. The proportion of Akt/p-Akt was compared according to the relative gray of the bands. (one-way ANOVA)

Moreover, it has been widely recognized, as a tumor-suppressive gene, PTEN loss was a frequent event that occurred in cancers and associated closely with tumor grading, staging, and prognosis, no exception for EOC [27]. As mentioned above, we found that miR-23a-3p was up-regulated in EOC, which was reverse to the its PTEN expression pattern. Moreover, bioinformatics prediction also proposed that miR-23a-3p could directly bind to PTEN (Fig. 5A). It urged us to deeply investigate whether PTEN was  the downstream target of miR-23a-3p to affect EOC's progression. So that, the dual-luciferase report analysis was performed. It revealed that co-transfection of miR-23a-3p mimic could weaken the luciferase activity of the PTEN 3′-UTR WT group. However, the luciferase activity of the PTEN 3′ UTR MUT group was not affected (Fig. 5B), which confirmed the previous hypothesis.

To investigate the regulatory effect of circRHOBTB3 on the miR-23a-3p’ target gene PTEN, we used Western blot assay to detect the expression of PTEN in each group. After transfection of the miR-23a-3p mimic, we observed that PTEN protein expression was significantly suppressed. (Fig. 5C), which further verified that PTEN was a downstream target of miR‐23a-3p. Consistently, western blot results demonstrated that the expression of PTEN significantly rose up when EOC cells were co-transfected with circRHOBTB3 OE plasmid and miR-23a-3p mimic, compared with the group transfected with miR-23a-3p mimics solely (Fig. 5C, D), which proved that circRHOBTB3 could effectively attenuate the decrease of PTEN induced by miR-23a-3p. These results raised great curiosity for us to investigate further the activation of AKT, an essential functional downstream molecular of PTEN. Compared with the control group, the p-Akt /AKT ratio in the miR-23a-3p group was significantly increased, indicating that AKT activation was enhanced. When circRHOBTB3 was co-transfected with miR-23a-3p, AKT activation was inhibited, which suggested that circRHOBTB3's regulation on PTEN was functional  (Fig. 5E, F).

### In vivo validations

**Fig. 6:**
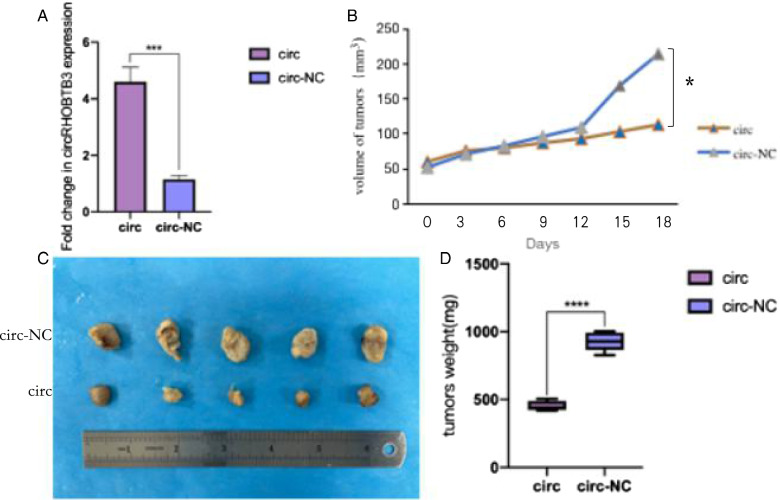
Over-expression of circRHOBTB3 reduces tumor growth in vivo. **A** the expression of circRHOBTB3 in the stable transgenic strains. **B** Volume growth curve of subcutaneously implanted tumor (Student’s t-test). **C** Anatomical display of subcutaneously implanted tumor in nude mice. **D** Comparision of the weight of subcutaneously implanted tumors between the control group with the circRHOBTB3 overexpressing group. (Student’s t-test)

To further investigate circRHOBTB3’s role in vivo, we constructed circRHOBTB3-OE/circRHOBTB3-NC stable transgenic strains (Fig. 6A) and injected them into mice subcutaneously to construct implantation models. It was observed that overexpression of circRHOBTB3 could significantly suppress the tumors' growth rate (Fig. 6B). After 18 days, subcutaneous tumors were dissected entirely, their size and weight were analyzed, the results revealed that circRHOBTB3 could significantly inhibit tumor growth in vivo (Fig. 6C, D).

## Discussion

CircRNAs exist ubiquitously in tissues, exosomes, and biofluids, including blood, urine, cerebrospinal fluid, and saliva [36, 37]. Due to their abundance, stability, and tissue-specificity in vivo, circRNAs can serve as efficient biomarkers to guide and monitor clinical work in the future [38]. CircRHOBTB3 has been suggested as an abnormally expressed endogenous ncRNA in ovarian cancer [14]. However, its inner mechanism was not explored. Giving it, we verified that circRHOBTB3 was downregulated and its expression was correlated with tumor stage, age, lymphatic invasion, and prognosis in EOC, thus, we proposed that circRHOBTB3 may play a profound role in regulating the tumor’s biological behavior. Subsequently, the functional assays were carried on to show its effect on proliferation, migration, cell cycle distribution, and apoptosis in SKOV3 and OVCAR8 cells. Previous studies have revealed that abundantly enriched circRNAs could act as miRNAs sponges in the cytoplasm [39–41]. We presumed that circRHOBTB3 could also act in that way. Given that miR-23a-3p overexpression was associated with poor survival and platinum resistance in OC patients [25]. And miR-23a-3p’s inhibitory effect of PTEN, a well-known tumor suppressor and a metabolic regulator [42] has been reported to engage in multiple biological dysregulations. We were inspired that miR-23a-3p/PTEN might be worth exploring as the downstream pathway for circRHOBTB3 in EOC. After checking on the bioinformatic database: Starbase, which predicted that circRHOBTB3/miR-23a-3p/PTEN might have direct combination sites with each other, we performed a series of experiments like the luciferase assay, FISH…, to validate our presumption. To our delight, They did have direct target sites with each other. Then, through the rescue experiments, we verified that by serving as the ceRNA of miR-23a-3p, circRHOBTB3 could inhibit proliferation, invasion, G1/S transition, while facilitate apoptosis and activate the PTEN/Akt pathway in EOC. We also succeed in invalidating our conclusion in xenograft tumor model.

However, our study has limitations due to the limited energy. First, in the present study, only 60 ovarian tissues were analyzed. Moreover, since the EOC tissues were so tightly bound to their adjacent tissues that it was very difficult to clearly separate them, we thus used the ovarian specimens from patients underwent the  bilateral adnexectomy for cervical cancers. What’s more, expression of PTEN and p-AKT in the subcutaneous tumor tissues could also be explored to further validate  our conclusion.

Secondly, we look forward to further research to explore why and how circRHOBTB3 was downregulated in OC, which will provide a deeper understanding of its inner molecular mechanism. For example, since DNA methylation typically reduces the expression of genes in cancer progression, it is of great interest to explore whether the low endogenous expression level of circRHOBTB3 in ovarian cancer tissues is epigenetically silenced by promoter hypermethylation [43]. In the pre-experimental phase, we detected an expression change of circRHOBTB3 when transfected si-METTL3 into SKOV3, but subsequently we came across some difficulties when trying to validate the hypermethylation of the circRHOBTB3's promoter. We believed that it deserved a more organized and integrated study to investigate deeply.

Furthermore, almost all studies at present only reported the character of circRHOBTB3 as miRNAs’ sponger in an Argonaute (AGO2)-dependent manner [44]; it remains to be explored whether there are other mechanisms for it to modulate gene expression. Recently, studies have shown that circRNAs can interact with regulatory RNA binding proteins (RBP) to influence the expression of their downstream mRNA [45]. Typically, RBP binds to the 3’UTR of the target mRNA to promote its stability, while circRNA's interaction with RBP could degrade it, thereby reducing the mRNA expression level [46]. For instance, the study done by J. Chen et al. revealed that circRHOBTB3 harbored a binding site of HuR, a ubiquitously expressed and functional RBP in CRC, and promoted β-Trcp1-mediated ubiquitination of HuR [11]. Moreover, our previous review summed up the cellular effects of another specific circular RNA: circHIPK3, including autophagy, angiogenesis, EMT/FMT, differentiation cytotoxic, and pyroptosis [47]. They can become promising directions in the further exploration on circRHOBTB3. For instance, Yang et al. demonstrated that induced by FUS, circRHOBTB3 could act as a novel autophagy promotive for pancreatic ductal adenocarcinoma (PDAC) through blocking Akt/mTOR pathway [13]. Finally, in addition to treasuring its value as a biomarker, its potential in conquering chemoresistance also deserves attention [ 48. ], since chemotherapy resistance remains one of the key reasons for the poor prognosis of ovarian cancer.

## Conclusions

In summary, current functional and mechanistic insights highlighted the predominant role of circRHOBTB3 in EOC's progression and their potential to serve as a novel prognosis biomarker. We also illustrated that circRHOBTB3-mediated miR-23a-3p/PTEN/Akt signaling could inhibit EOC’s malignant progression, providing a promising treatment strategy for treating it. but whether circRHOBTB3 can be adhibited as an effective target in the clinic still depends on deeper investigations.

## Supplementary Information

**Additional file 1.**

**Additional file 2.**

**Additional file 3.**

**Additional file 4.**
