# Supplementary material for: The molecular mechanism of circRHOBTB3 inhibits the proliferation and invasion of epithelial ovarian cancer by serving as the ceRNA of miR-23a-3p
Source: J Ovarian Res. 2022 Jun 1;15:66. doi: 10.1186/s13048-022-00979-1 (PMC9158168; doi:10.1186/s13048-022-00979-1)
Supplement: Supplementary file 1 — Additional file 1. [file 13048_2022_979_MOESM1_ESM.zip › circRHOBTB3-OE/circRHOBTB3-OE.vectors.pdf]

PRODUCT DATA SHEET

Public Protein/Plasmid Library

pLenti-CMV-hsa_circ_0007444-GFP-Puro

Cat No.: PPL02463-4a   Quantity: Please refer to the label (tube)

Gene/insert name: hsa_circ_0007444
Insert size: 479bp
Species: Homo sapiens (human)
Gene ID: N/A
Accessions: N/A
Vector backbone: pLenti-CMV-circRNA-GFP-Puro
Tag: None
Backbone manufacturer: N/A
Vector type: Mammalian Expression, Lentiviral
Backbone size w/o insert: 9177bp
Cloning site 5': EcoR I
Site destroyed during cloning: NO
Cloning site 3': BamH I
Site destroyed during cloning: NO
Bacterial resistance(s): Ampicillin
Growth strain(s): Stbl3
Growth temperature: 37 ℃
High or low copy: High Copy
Selectable markers: Puromycin
Plasmid description: Lentiviral expression vector of Homo sapiens hsa_circ_0007444
Alt name: N/A

Map:

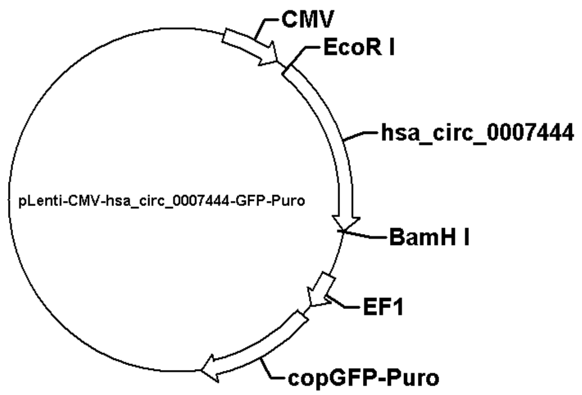

Buffer :
Sterile distilled water.

Storage:
The product at -20℃ for up to 24 months.

Note:
For research use only, not for use in diagnostic procedure.

Public Protein/Plasmid Library
No 9, weidi road Qixia District Nanjing, 210046,
Tel: 0086-025-68037686

Geneppl technology, co, Ltd.
Email: order@geneppl.com
www.geneppl.com
