# Supplementary figures and images for: The molecular mechanism of circRHOBTB3 inhibits the proliferation and invasion of epithelial ovarian cancer by serving as the ceRNA of miR-23a-3p
Source: J Ovarian Res. 2022 Jun 1;15:66. doi: 10.1186/s13048-022-00979-1 (PMC9158168; doi:10.1186/s13048-022-00979-1)

pHBLV-CMV-ZsGreen-T2A-Puro


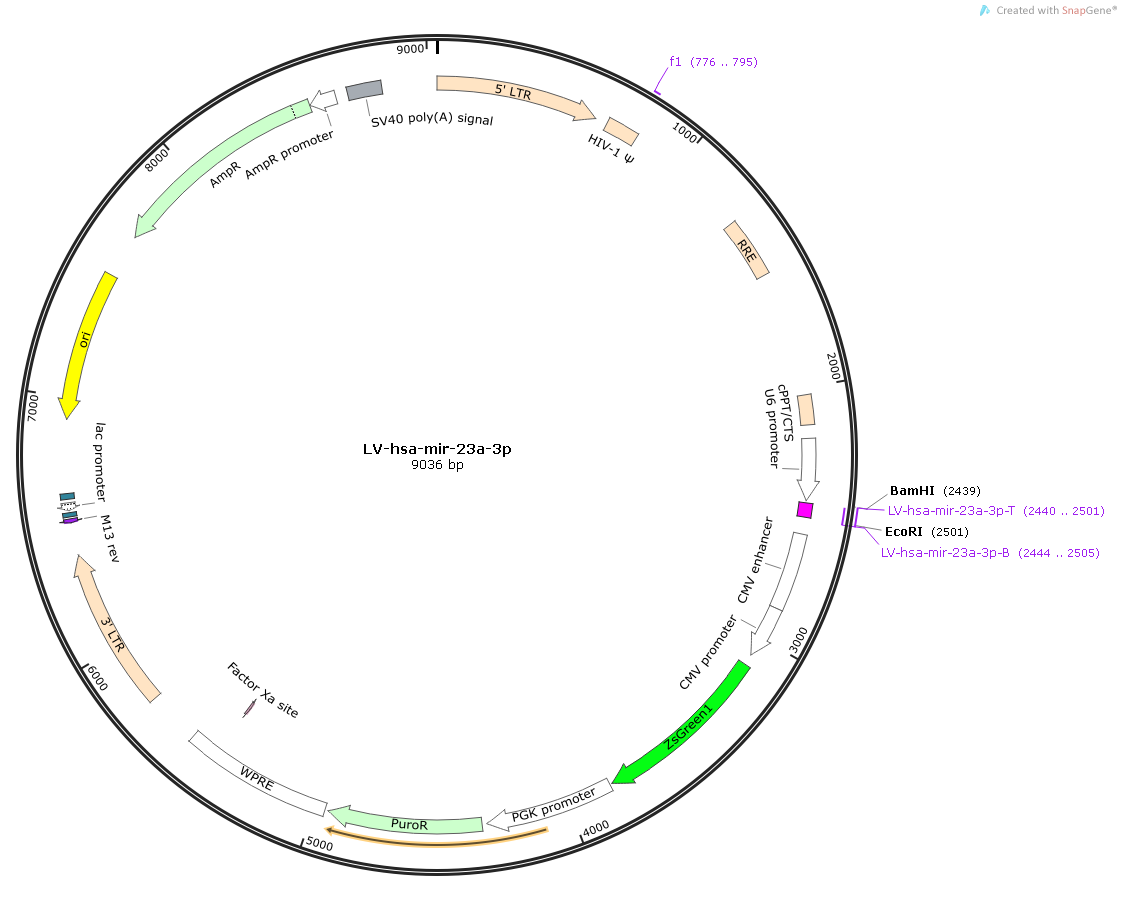


>hsa-miR-23a-3p MIMAT0000078


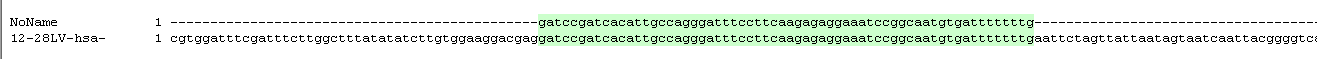

Supplement: Supplementary file 1 — Additional file 1. [file 13048_2022_979_MOESM1_ESM.zip › miR-23a-3p.docx]

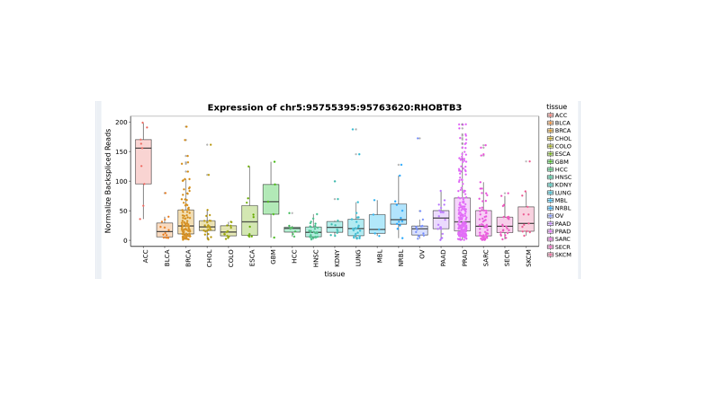

Supplement: Supplementary file 3 — Additional file 3. [file 13048_2022_979_MOESM3_ESM.zip › 3.tiff]

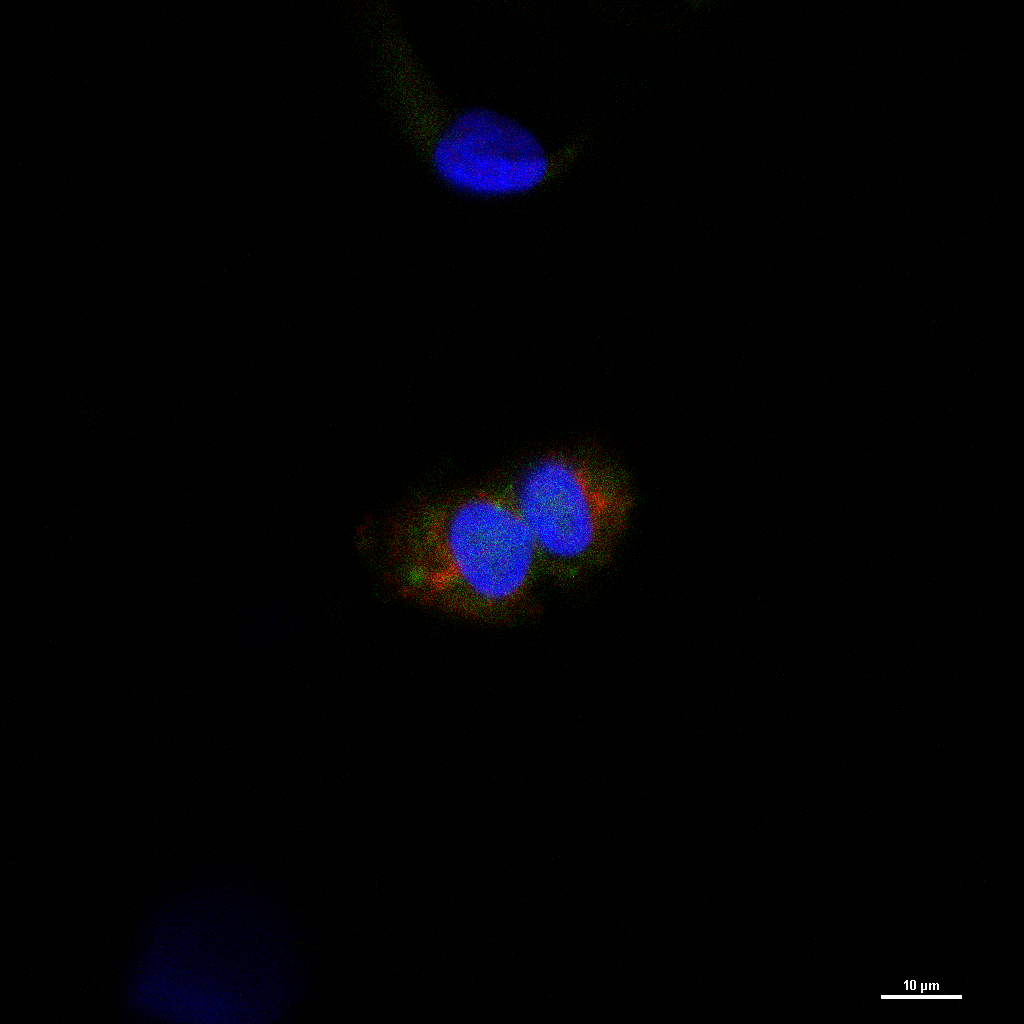

Supplement: Supplementary file 4 — Additional file 4. [file 13048_2022_979_MOESM4_ESM.zip › cell-climbing FISH/cell circ-0007444+mir-23a-3p(red) 1000-1.tif]

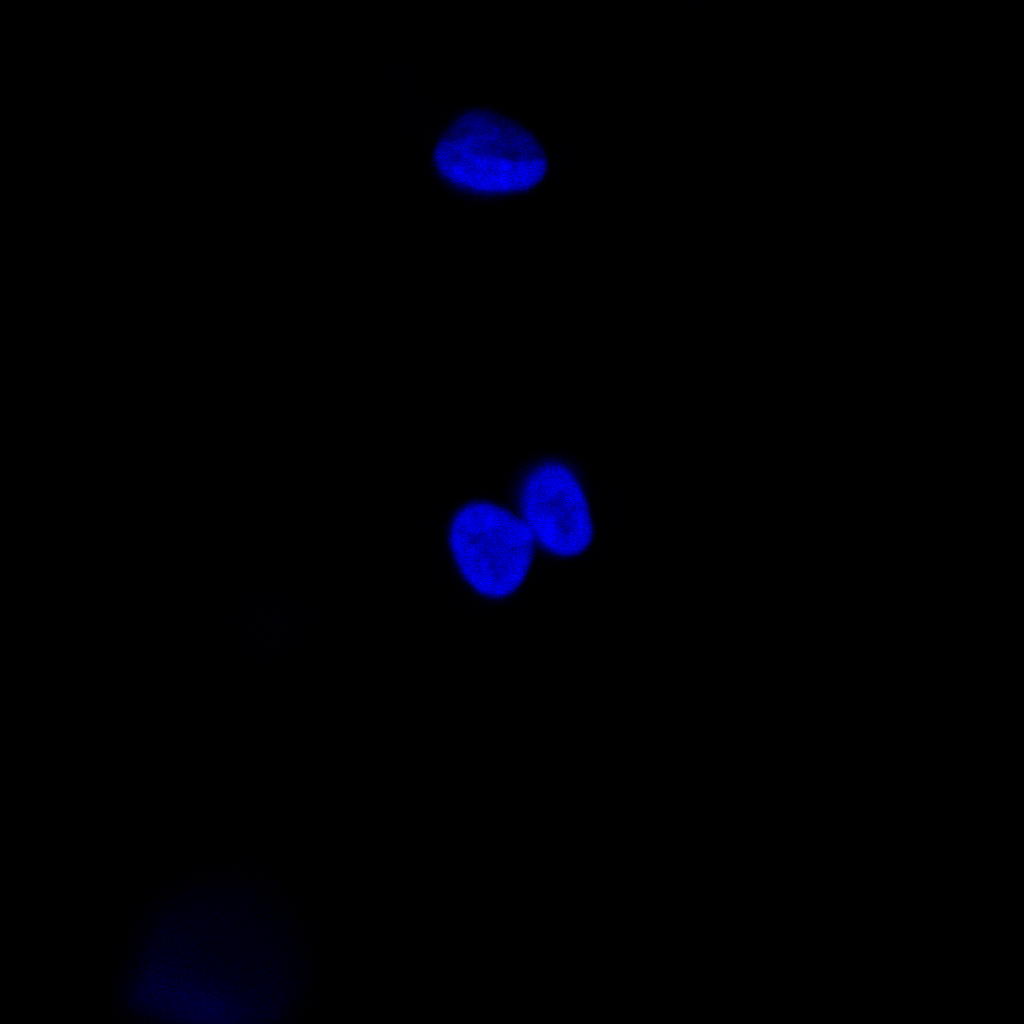

Supplement: Supplementary file 4 — Additional file 4. [file 13048_2022_979_MOESM4_ESM.zip › cell-climbing FISH/cell circ-0007444+mir-23a-3p(red) 1000-1c1.tif]

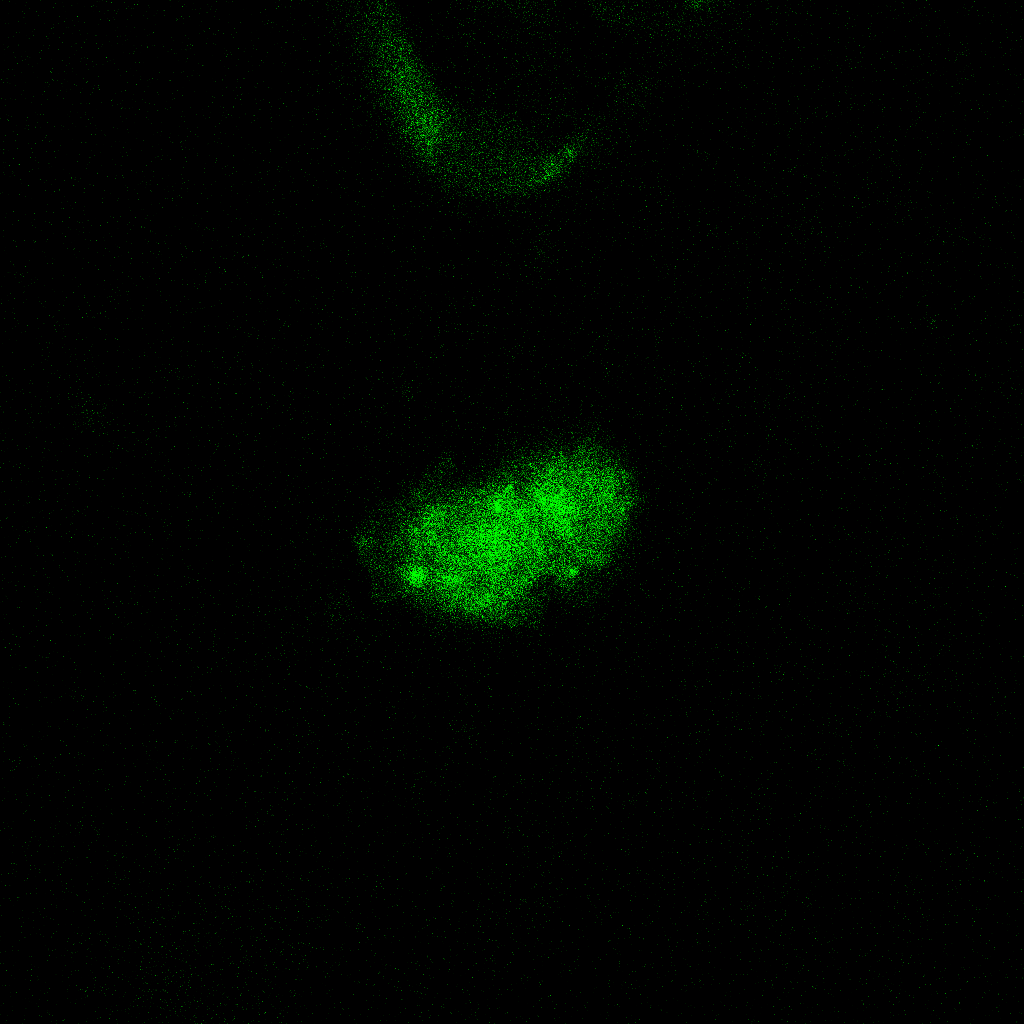

Supplement: Supplementary file 4 — Additional file 4. [file 13048_2022_979_MOESM4_ESM.zip › cell-climbing FISH/cell circ-0007444+mir-23a-3p(red) 1000-1c2.tif]

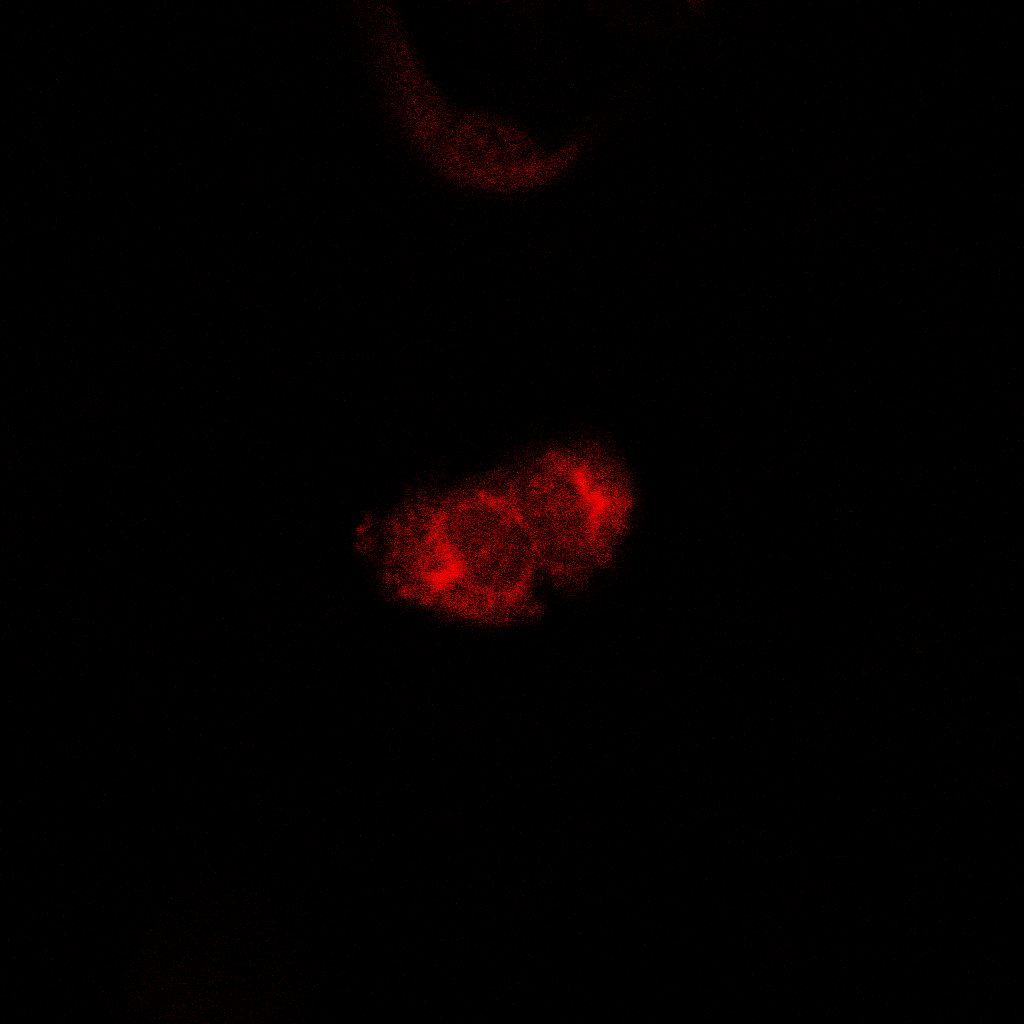

Supplement: Supplementary file 4 — Additional file 4. [file 13048_2022_979_MOESM4_ESM.zip › cell-climbing FISH/cell circ-0007444+mir-23a-3p(red) 1000-1c3.tif]

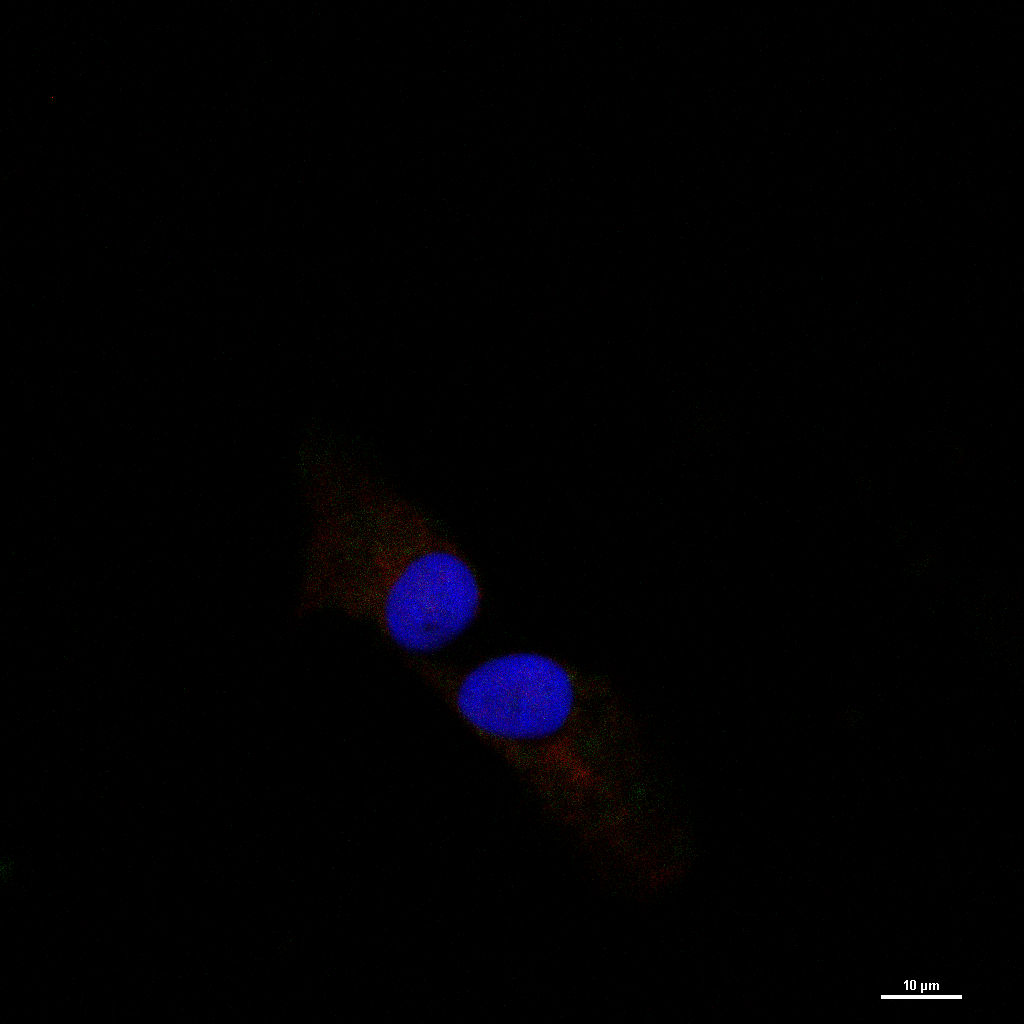

Supplement: Supplementary file 4 — Additional file 4. [file 13048_2022_979_MOESM4_ESM.zip › cell-climbing FISH/cell circ-0007444+mir-23a-3p(red) 1000-2.tif]

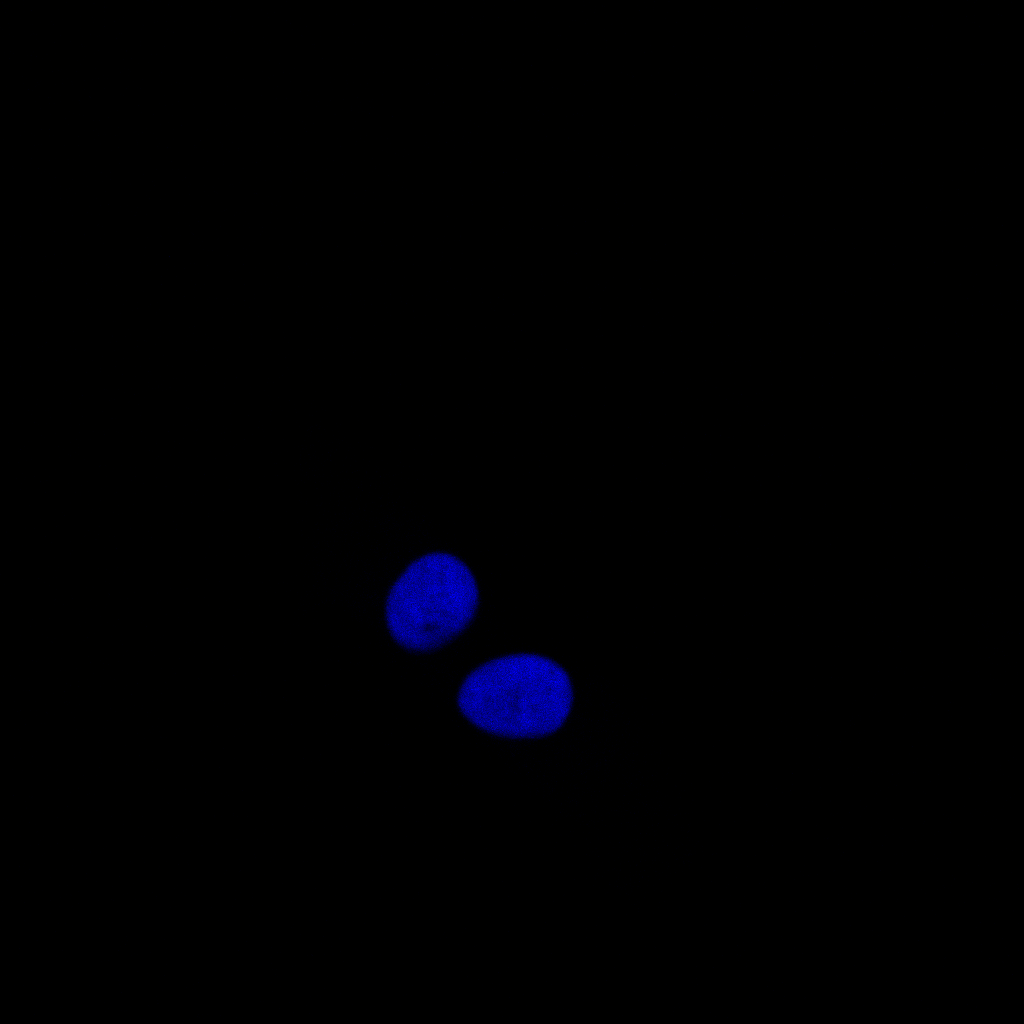

Supplement: Supplementary file 4 — Additional file 4. [file 13048_2022_979_MOESM4_ESM.zip › cell-climbing FISH/cell circ-0007444+mir-23a-3p(red) 1000-2c1.tif]

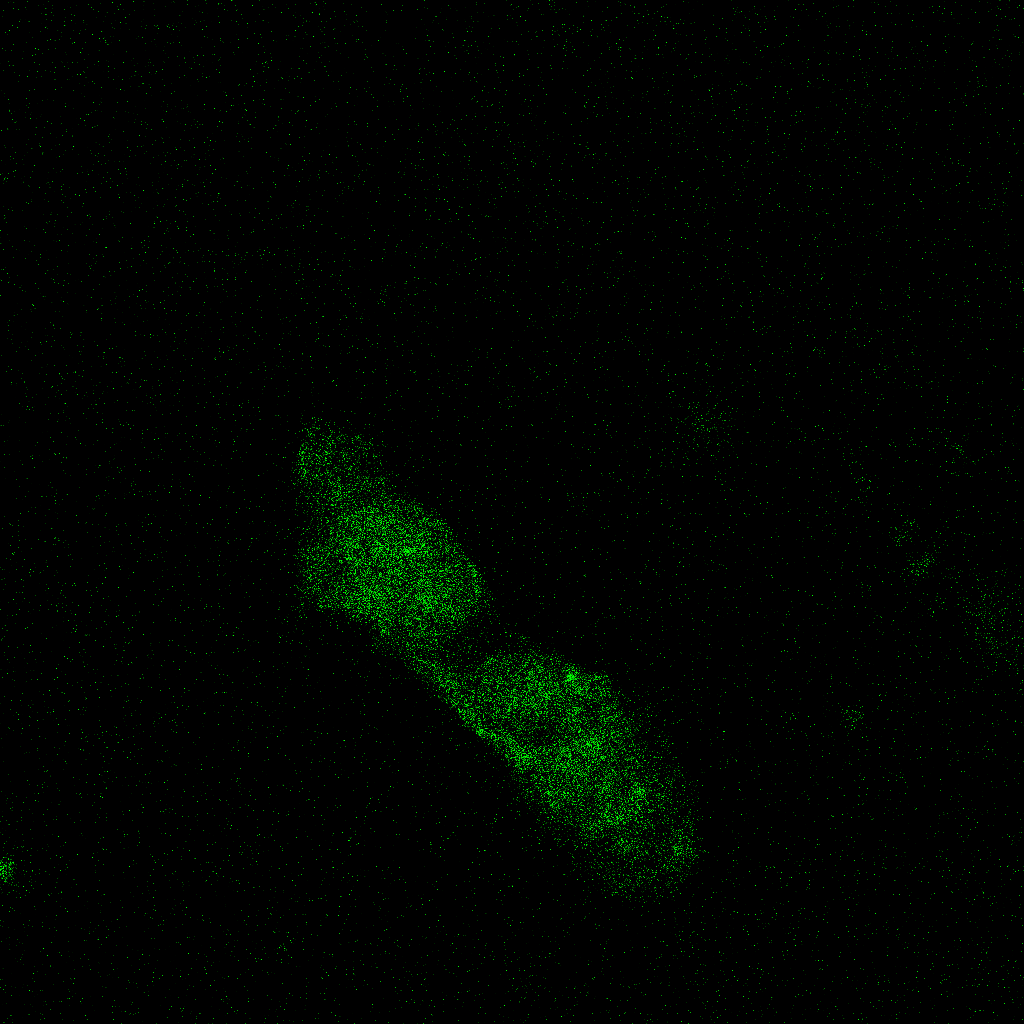

Supplement: Supplementary file 4 — Additional file 4. [file 13048_2022_979_MOESM4_ESM.zip › cell-climbing FISH/cell circ-0007444+mir-23a-3p(red) 1000-2c2.tif]

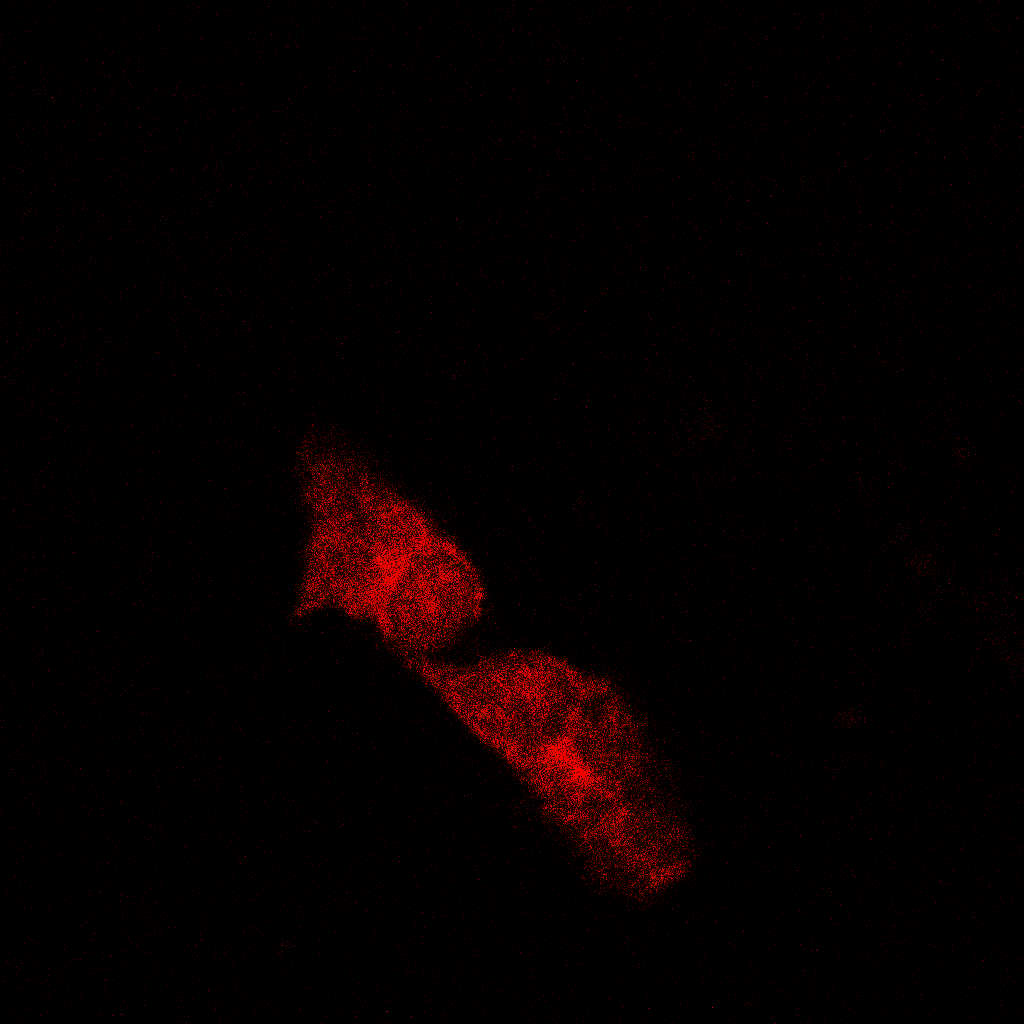

Supplement: Supplementary file 4 — Additional file 4. [file 13048_2022_979_MOESM4_ESM.zip › cell-climbing FISH/cell circ-0007444+mir-23a-3p(red) 1000-2c3.tif]

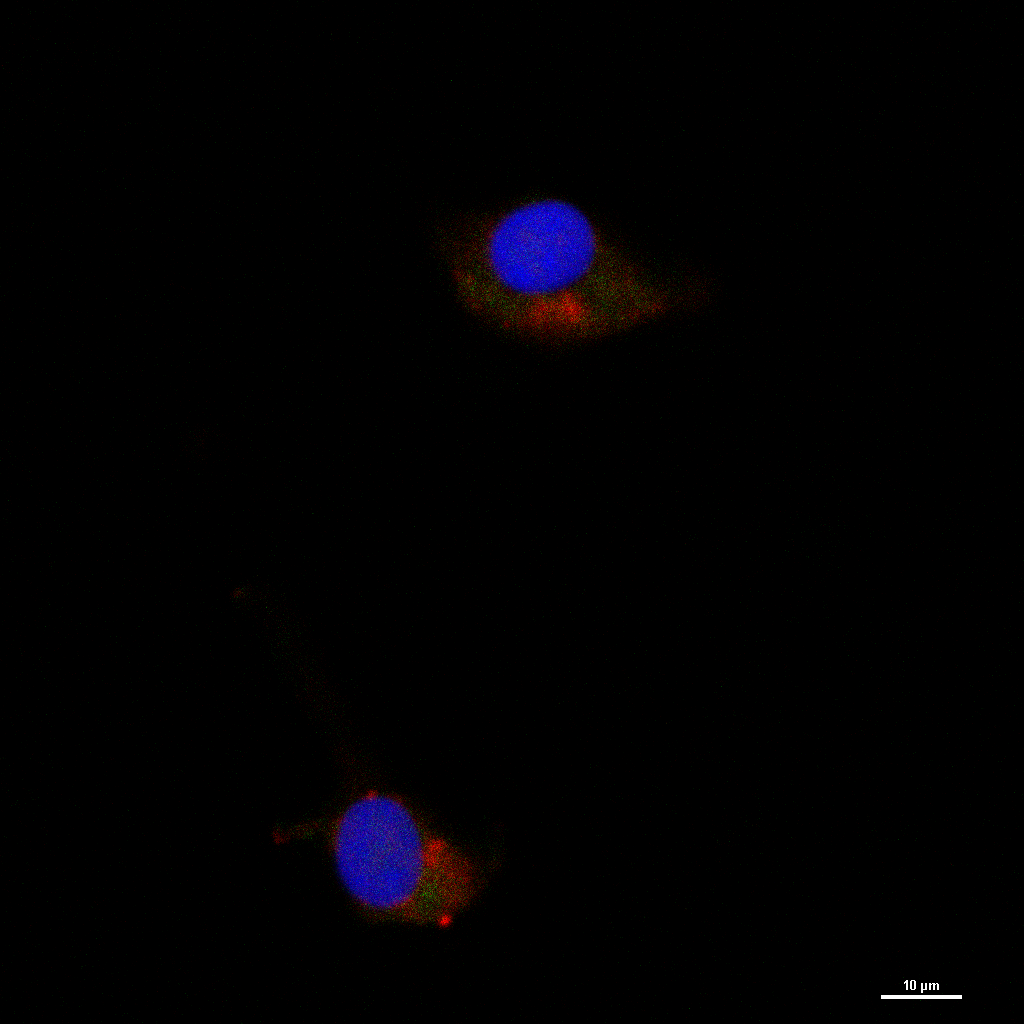

Supplement: Supplementary file 4 — Additional file 4. [file 13048_2022_979_MOESM4_ESM.zip › cell-climbing FISH/cell circ-0007444+mir-23a-3p(red) 1000-3.tif]

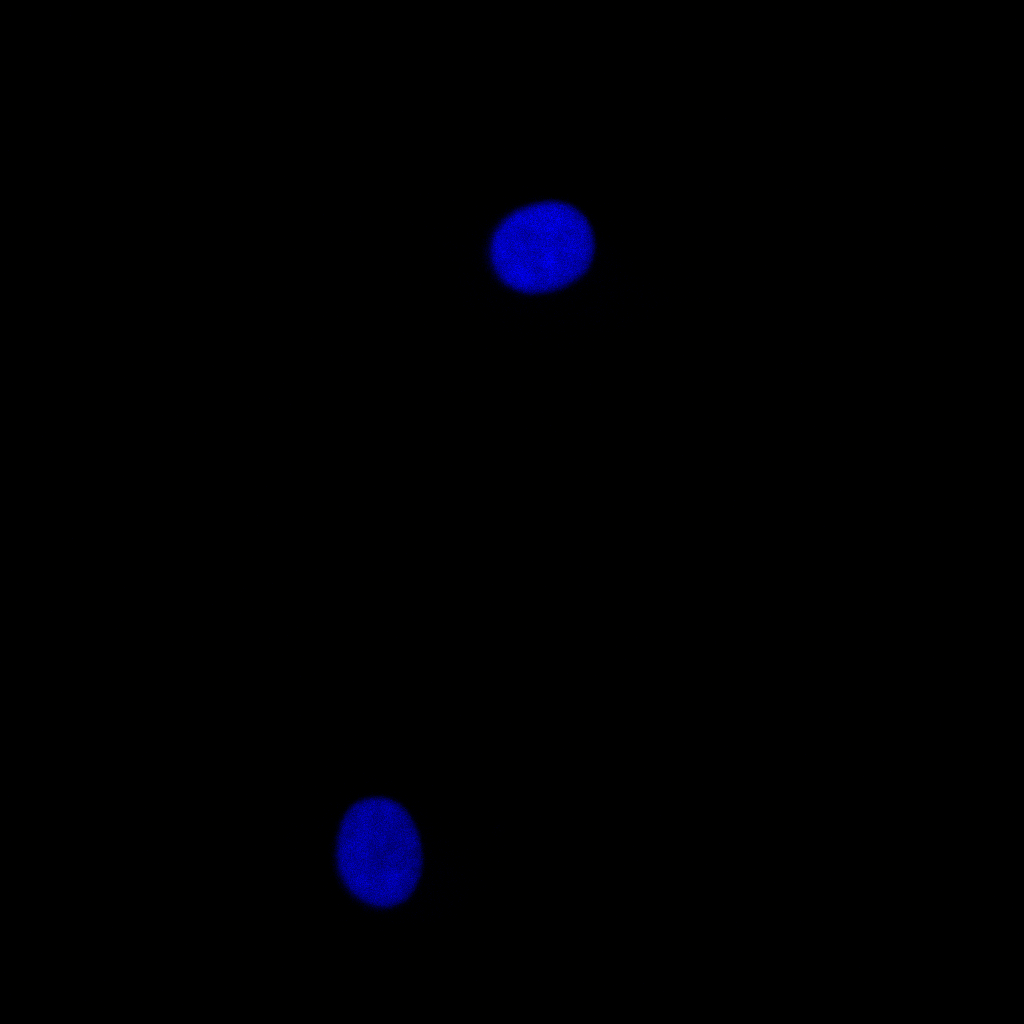

Supplement: Supplementary file 4 — Additional file 4. [file 13048_2022_979_MOESM4_ESM.zip › cell-climbing FISH/cell circ-0007444+mir-23a-3p(red) 1000-3c1.tif]

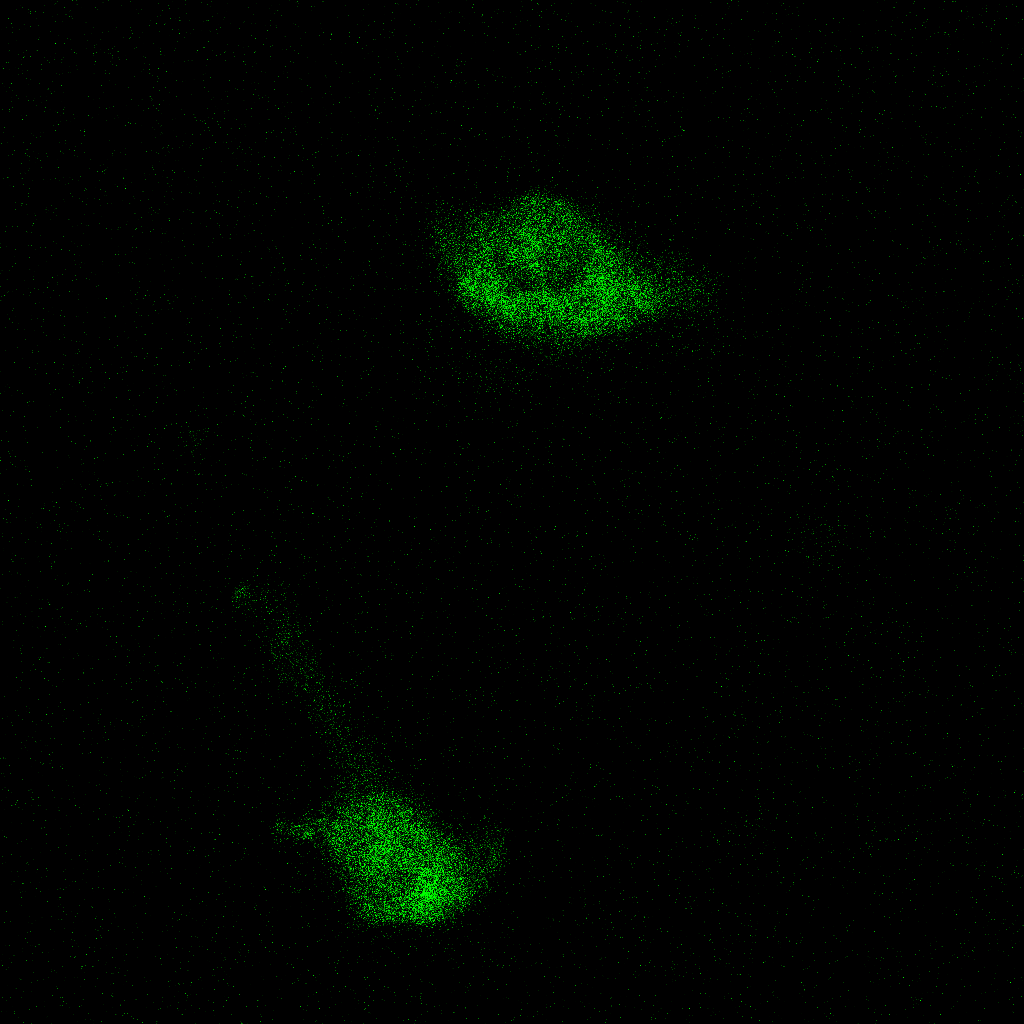

Supplement: Supplementary file 4 — Additional file 4. [file 13048_2022_979_MOESM4_ESM.zip › cell-climbing FISH/cell circ-0007444+mir-23a-3p(red) 1000-3c2.tif]

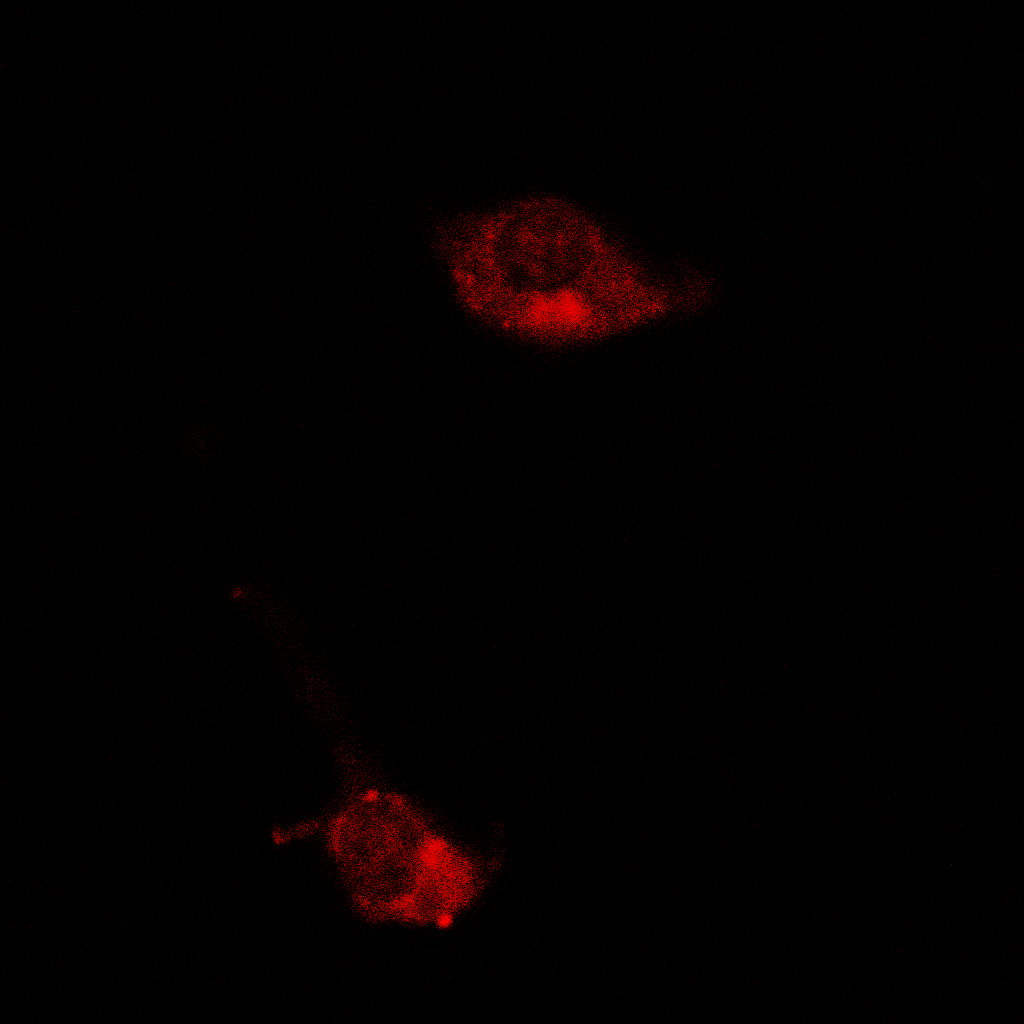

Supplement: Supplementary file 4 — Additional file 4. [file 13048_2022_979_MOESM4_ESM.zip › cell-climbing FISH/cell circ-0007444+mir-23a-3p(red) 1000-3c3.tif]

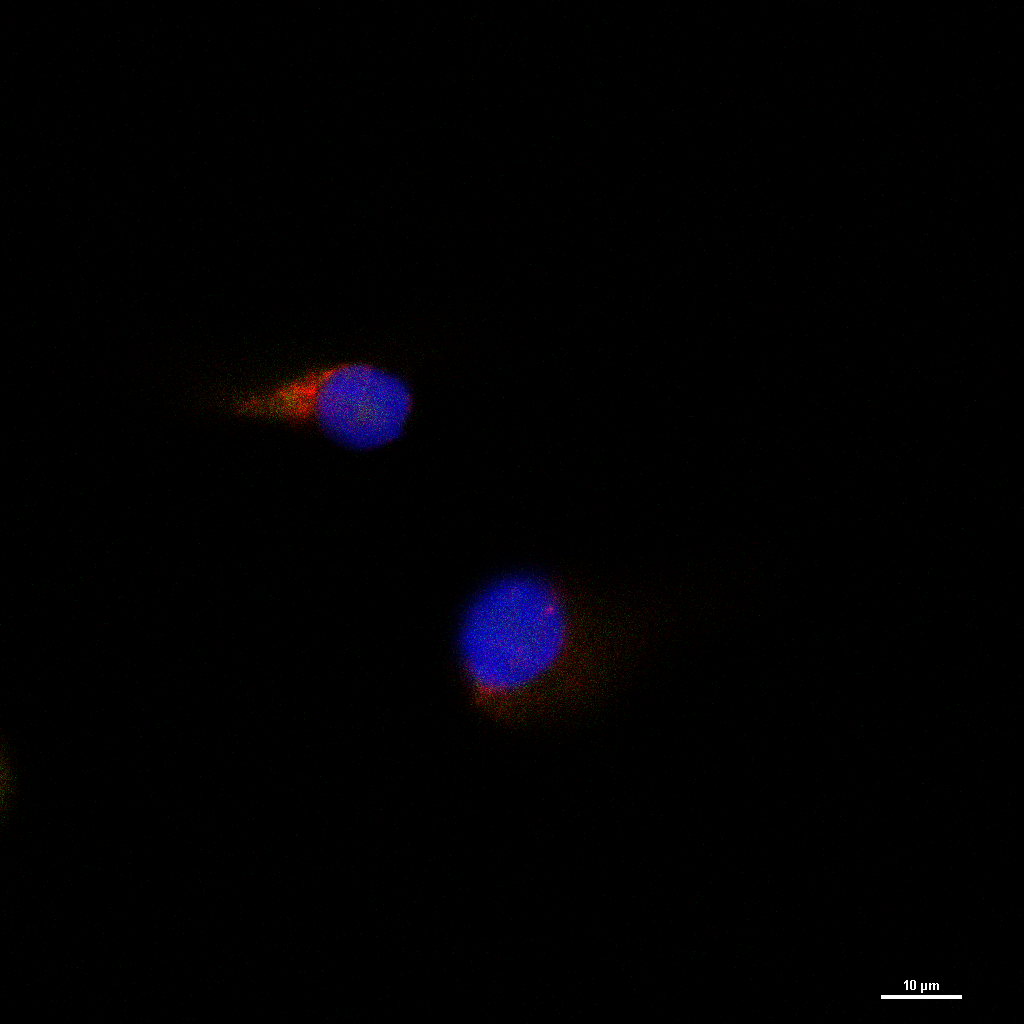

Supplement: Supplementary file 4 — Additional file 4. [file 13048_2022_979_MOESM4_ESM.zip › cell-climbing FISH/cell circ-0007444+mir-23a-3p(red) 1000-4.tif]

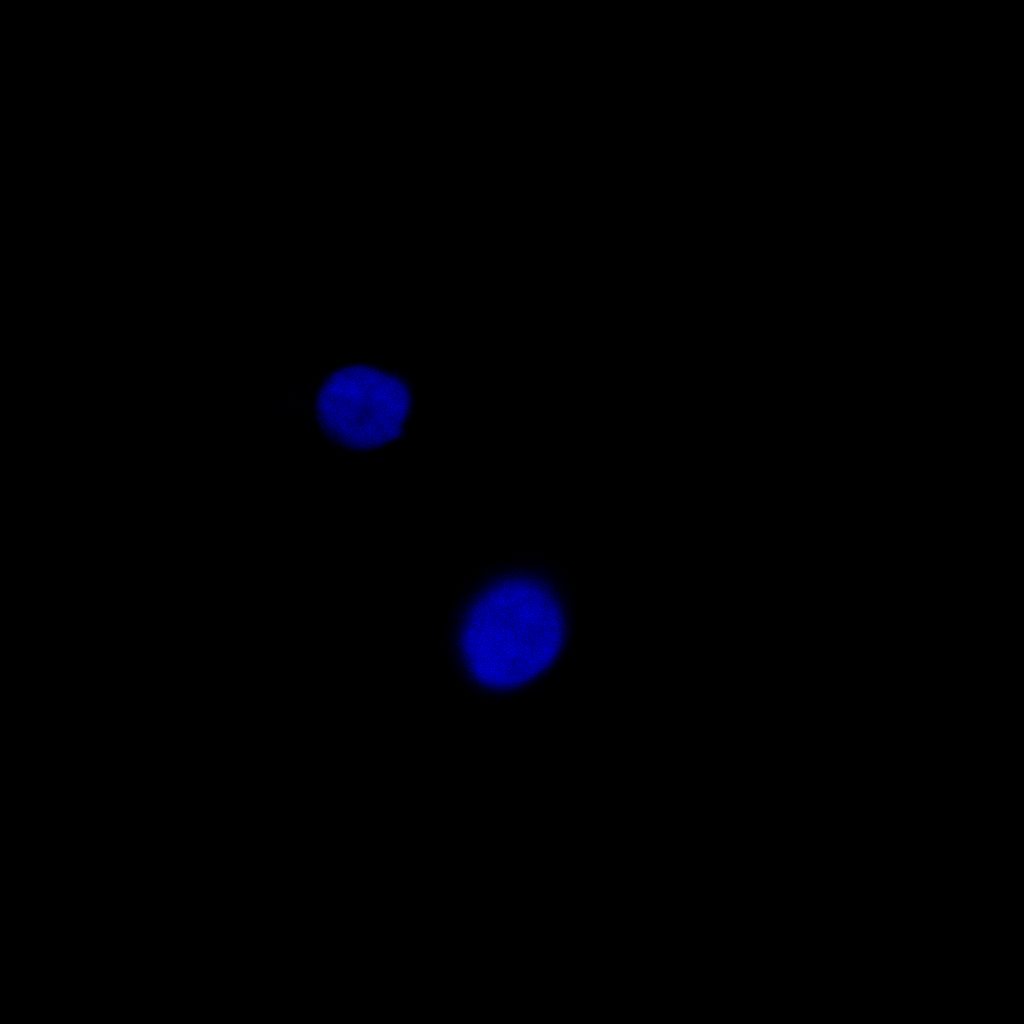

Supplement: Supplementary file 4 — Additional file 4. [file 13048_2022_979_MOESM4_ESM.zip › cell-climbing FISH/cell circ-0007444+mir-23a-3p(red) 1000-4c1.tif]

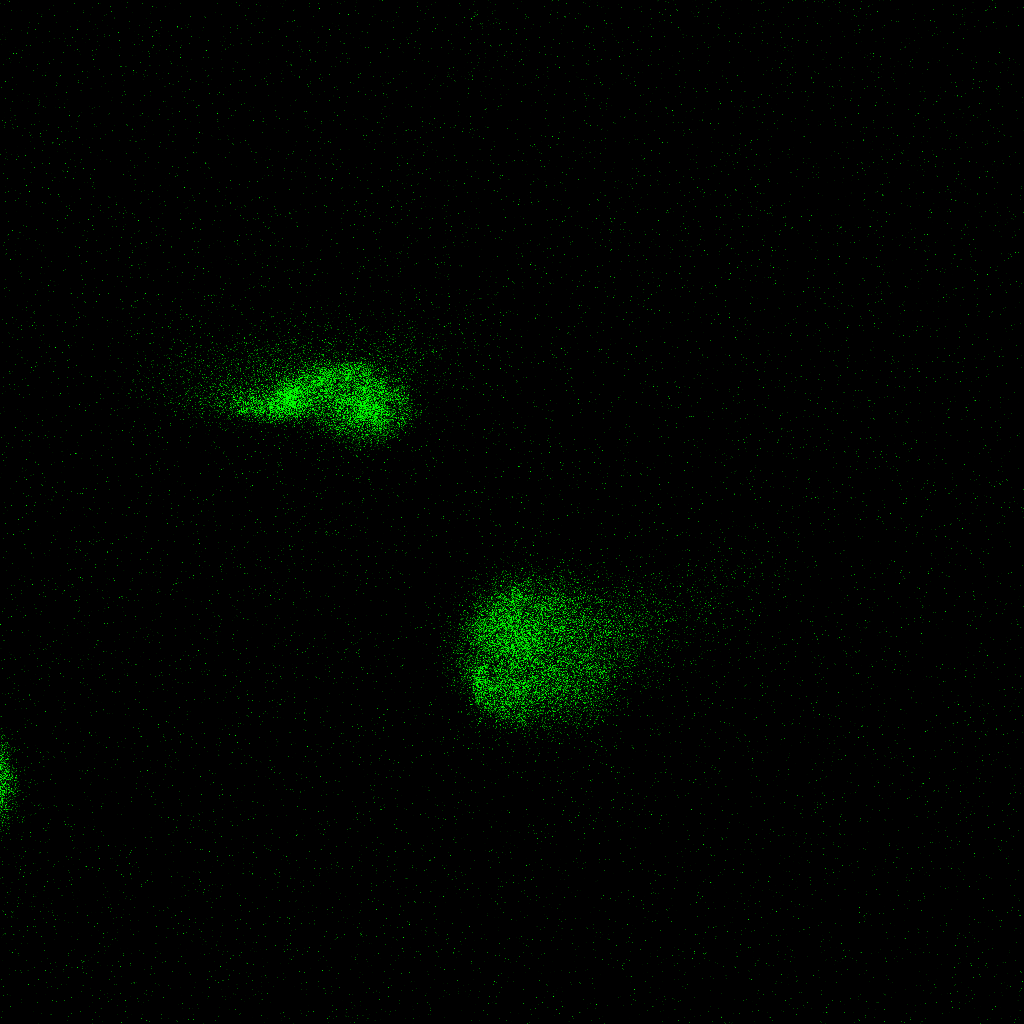

Supplement: Supplementary file 4 — Additional file 4. [file 13048_2022_979_MOESM4_ESM.zip › cell-climbing FISH/cell circ-0007444+mir-23a-3p(red) 1000-4c2.tif]

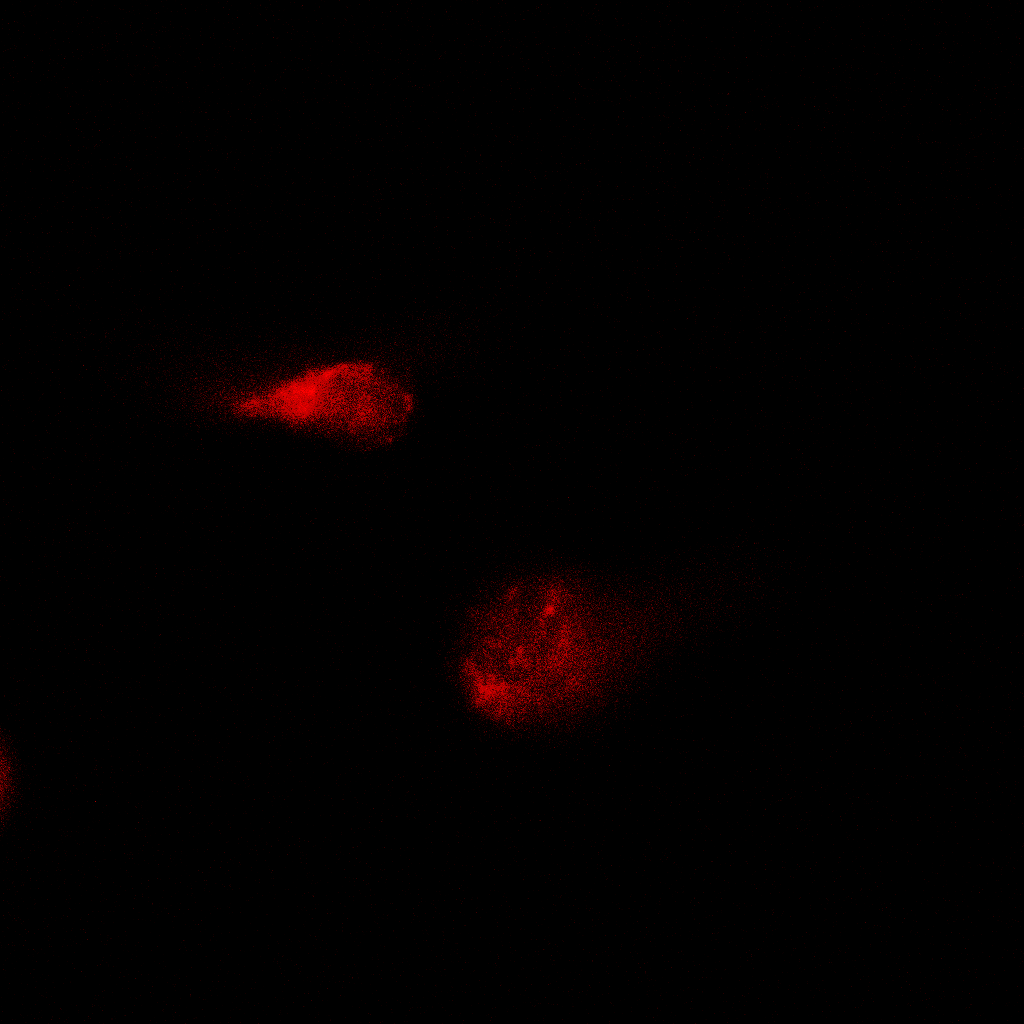

Supplement: Supplementary file 4 — Additional file 4. [file 13048_2022_979_MOESM4_ESM.zip › cell-climbing FISH/cell circ-0007444+mir-23a-3p(red) 1000-4c3.tif]

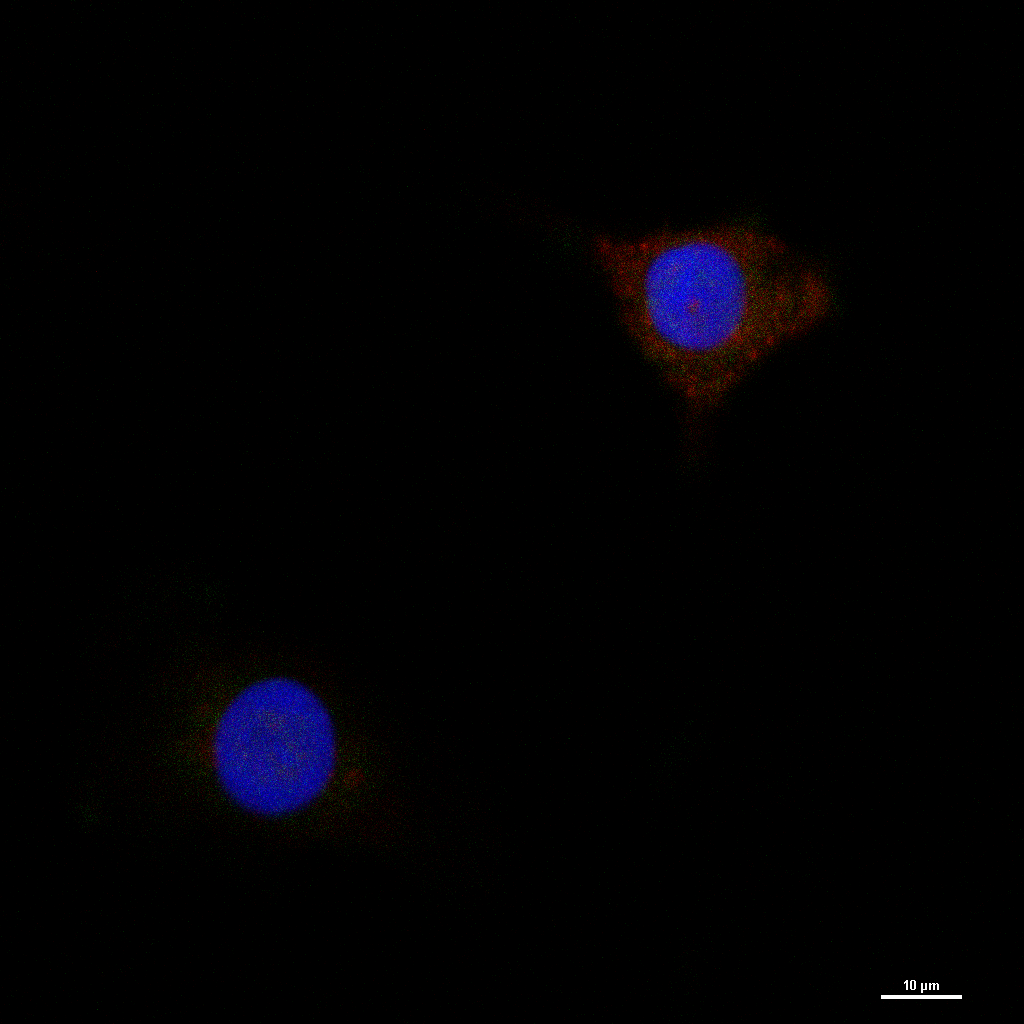

Supplement: Supplementary file 4 — Additional file 4. [file 13048_2022_979_MOESM4_ESM.zip › cell-climbing FISH/cell circ-0007444+mir-23a-3p(red) 1000-5.tif]

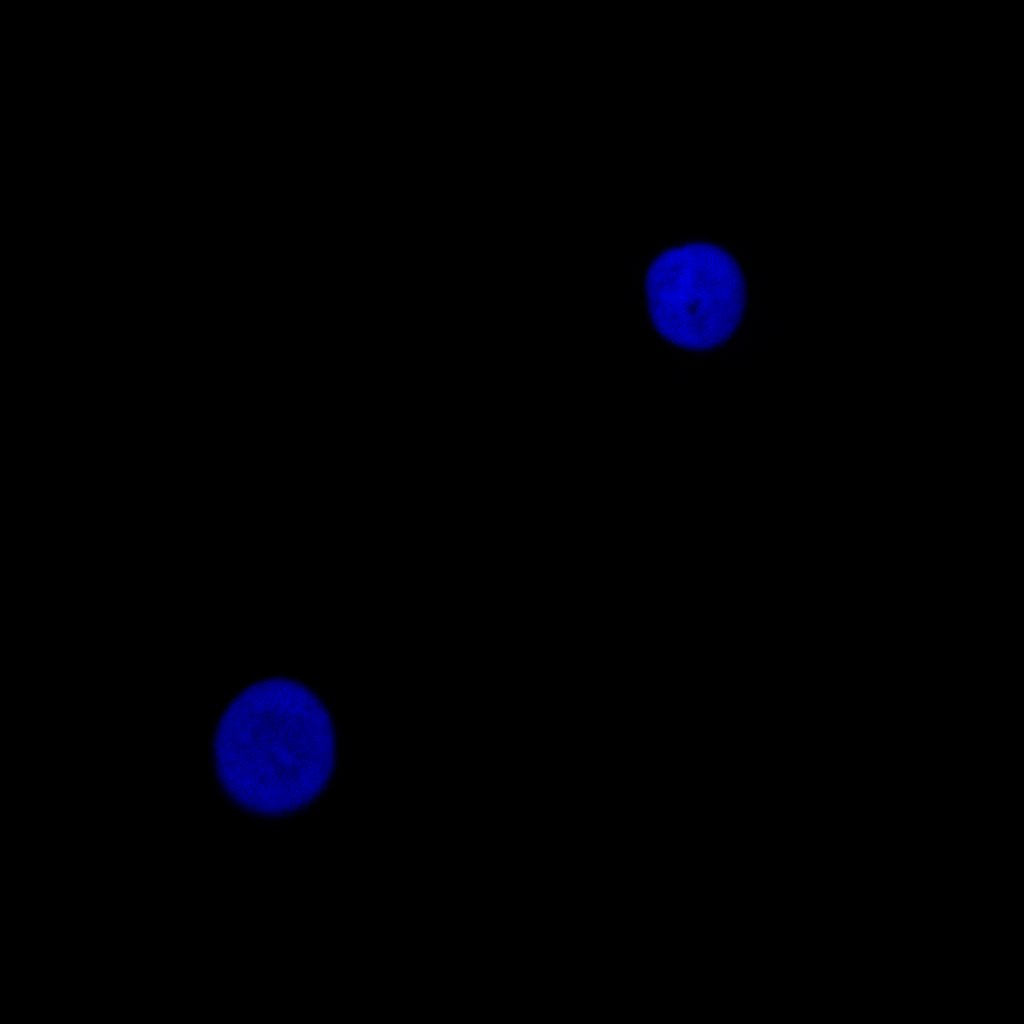

Supplement: Supplementary file 4 — Additional file 4. [file 13048_2022_979_MOESM4_ESM.zip › cell-climbing FISH/cell circ-0007444+mir-23a-3p(red) 1000-5c1.tif]

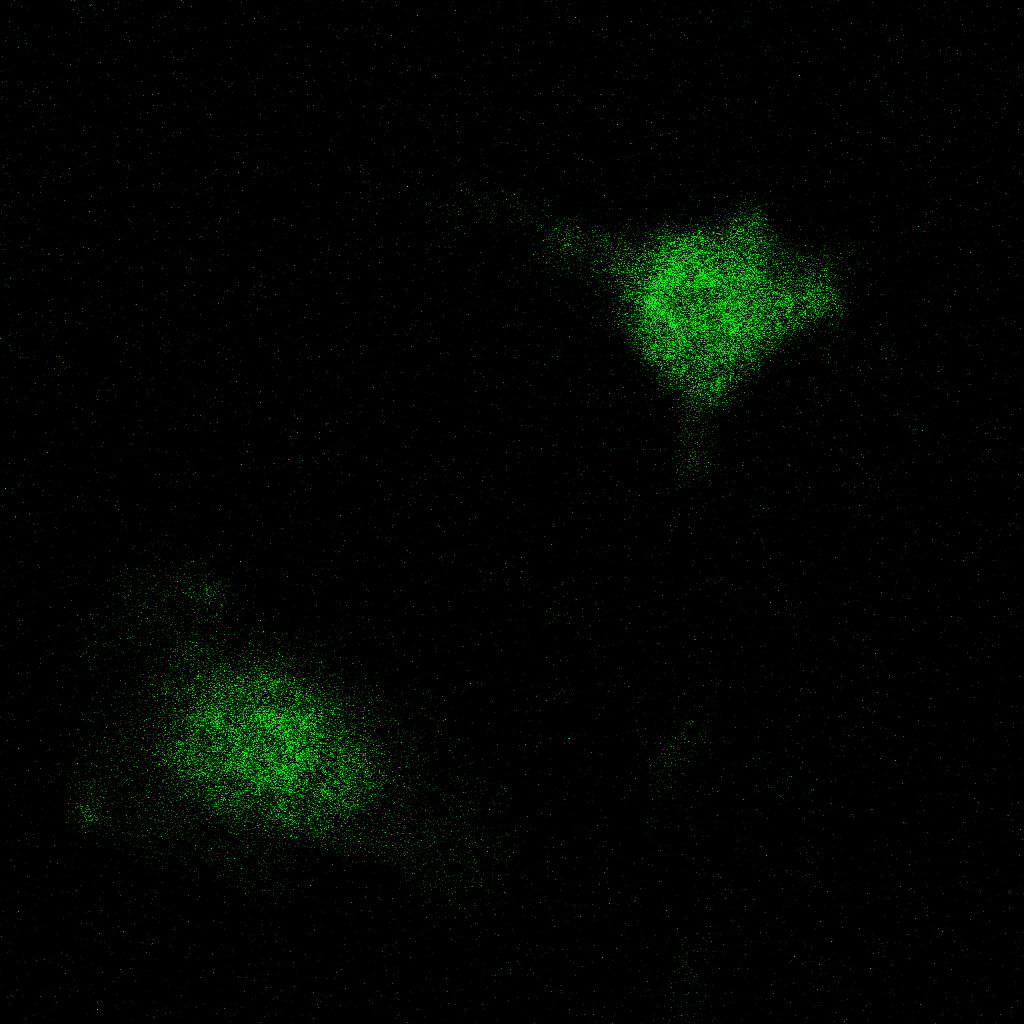

Supplement: Supplementary file 4 — Additional file 4. [file 13048_2022_979_MOESM4_ESM.zip › cell-climbing FISH/cell circ-0007444+mir-23a-3p(red) 1000-5c2.tif]

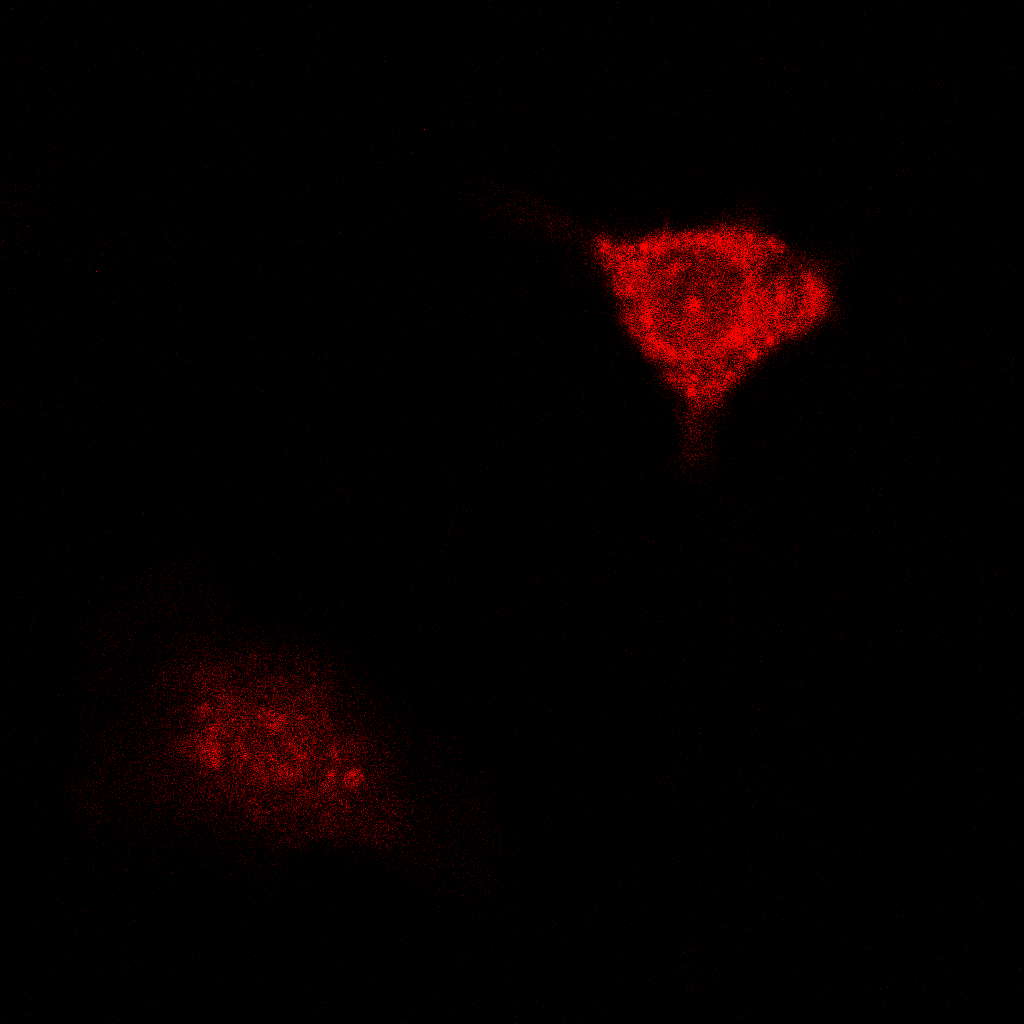

Supplement: Supplementary file 4 — Additional file 4. [file 13048_2022_979_MOESM4_ESM.zip › cell-climbing FISH/cell circ-0007444+mir-23a-3p(red) 1000-5c3.tif]

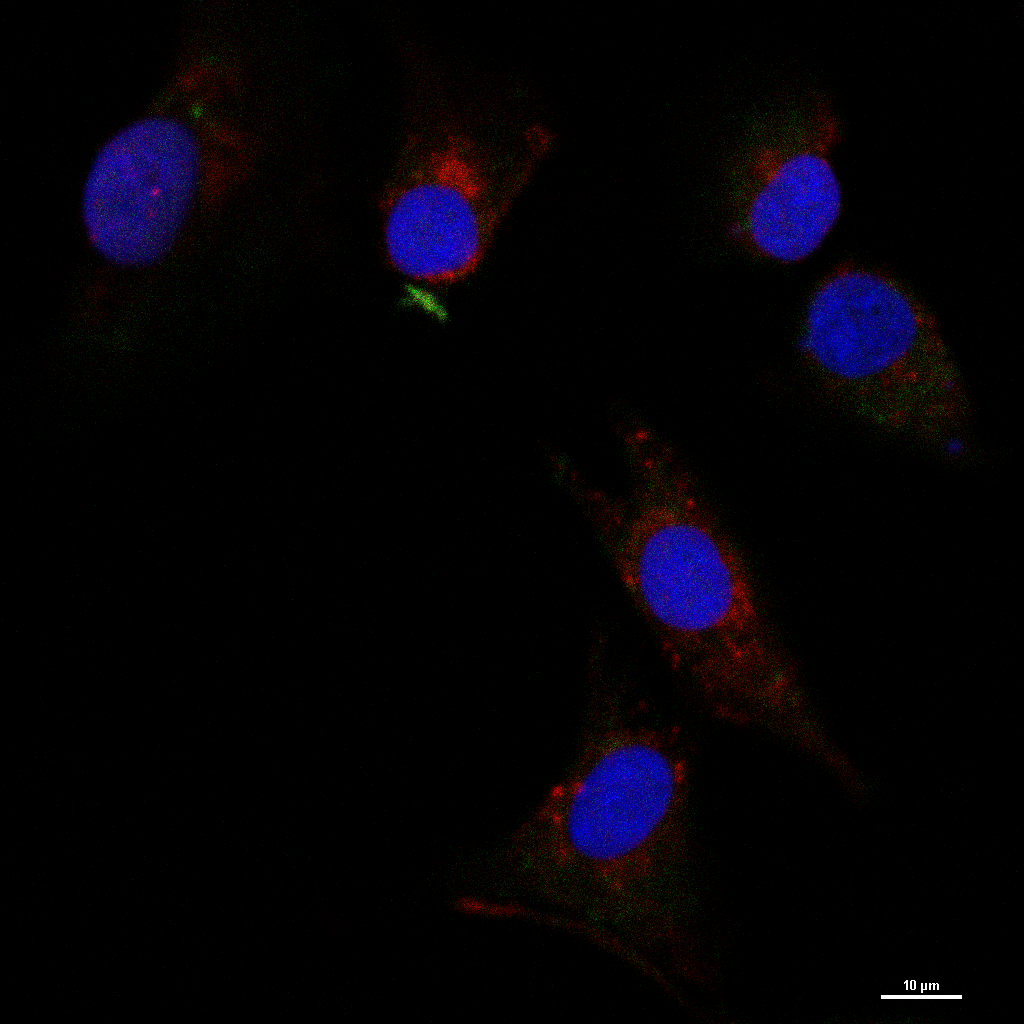

Supplement: Supplementary file 4 — Additional file 4. [file 13048_2022_979_MOESM4_ESM.zip › cell-climbing FISH/cell circ-0007444+mir-23a-3p(red) 1000-6.tif]

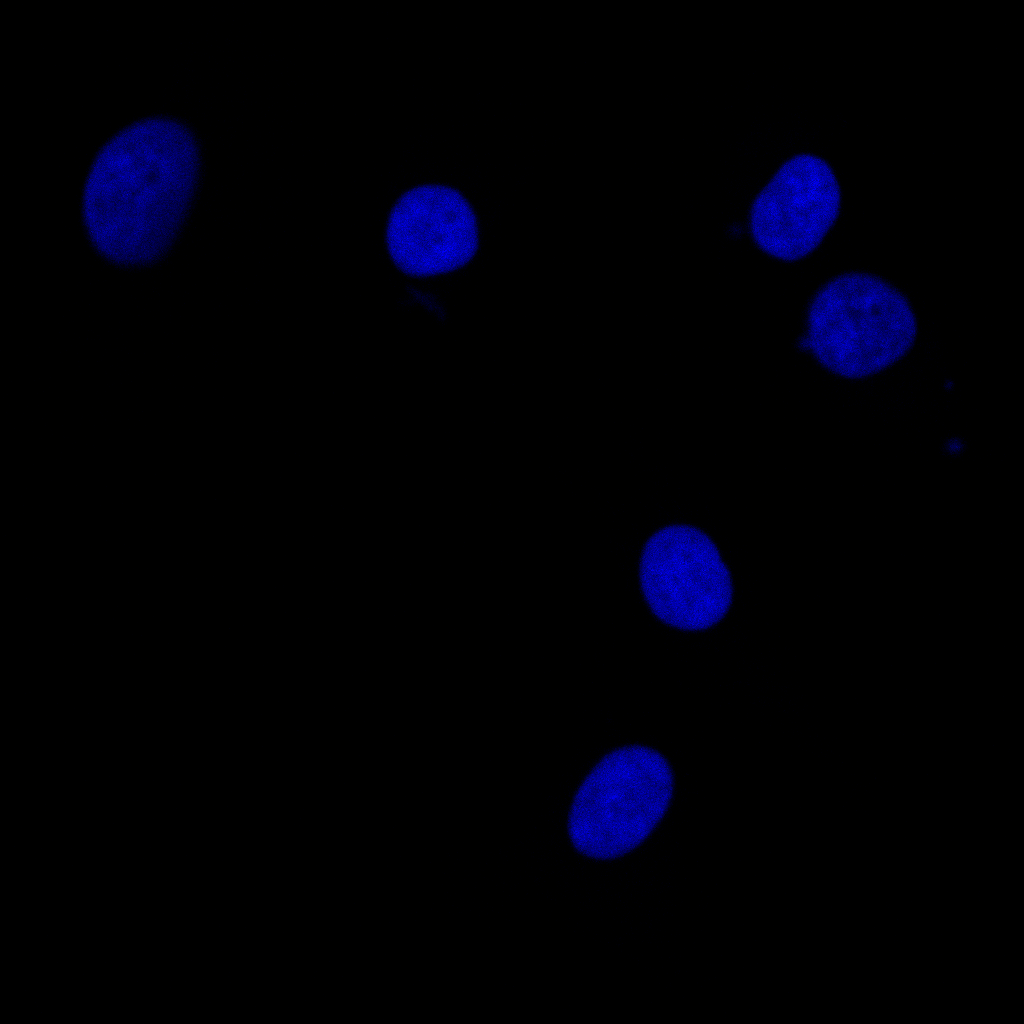

Supplement: Supplementary file 4 — Additional file 4. [file 13048_2022_979_MOESM4_ESM.zip › cell-climbing FISH/cell circ-0007444+mir-23a-3p(red) 1000-6c1.tif]

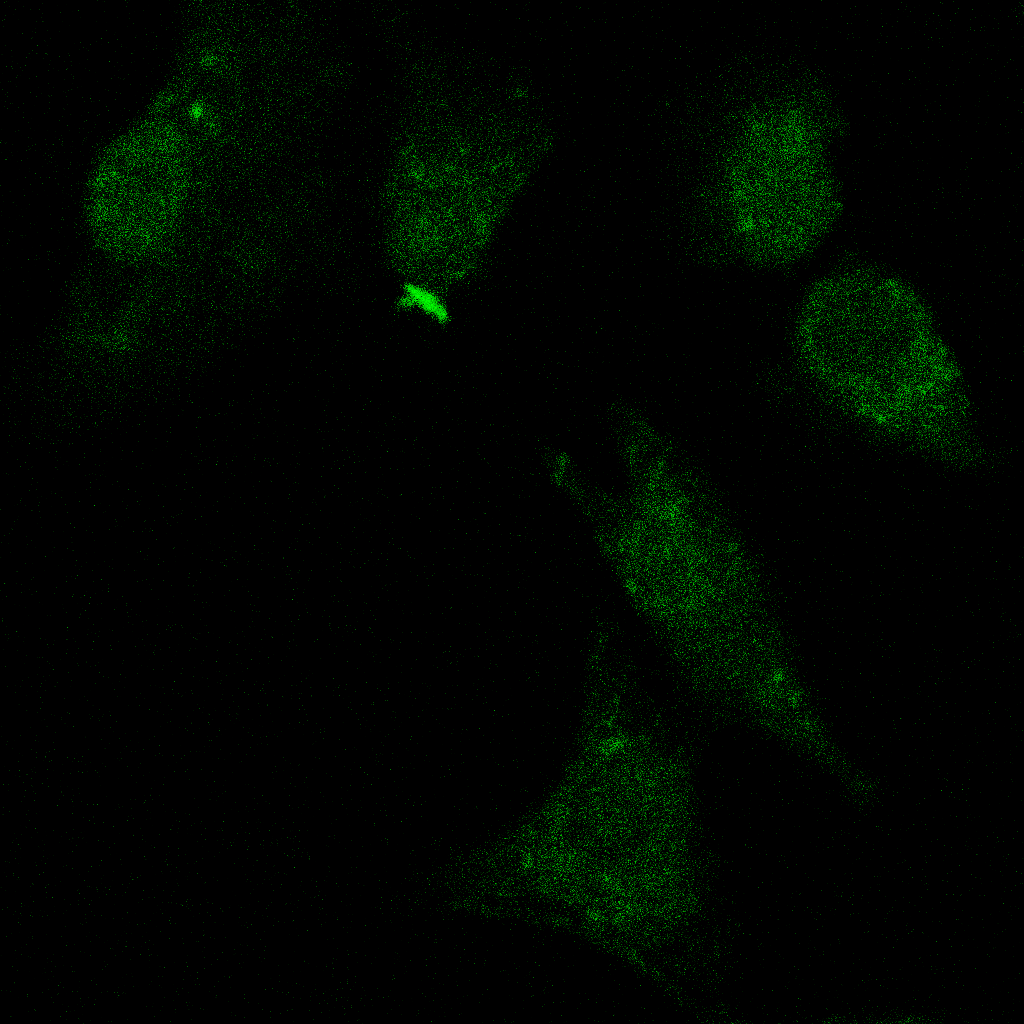

Supplement: Supplementary file 4 — Additional file 4. [file 13048_2022_979_MOESM4_ESM.zip › cell-climbing FISH/cell circ-0007444+mir-23a-3p(red) 1000-6c2.tif]

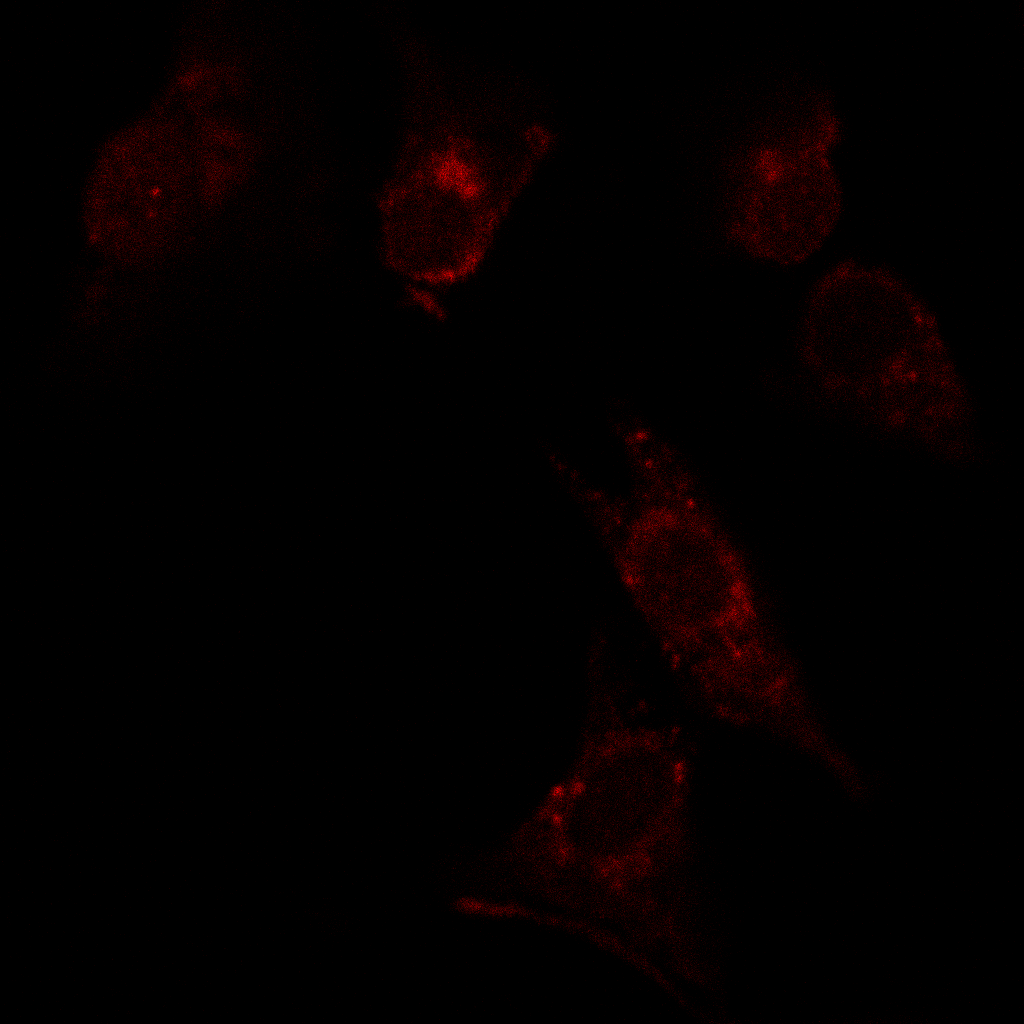

Supplement: Supplementary file 4 — Additional file 4. [file 13048_2022_979_MOESM4_ESM.zip › cell-climbing FISH/cell circ-0007444+mir-23a-3p(red) 1000-6c3.tif]

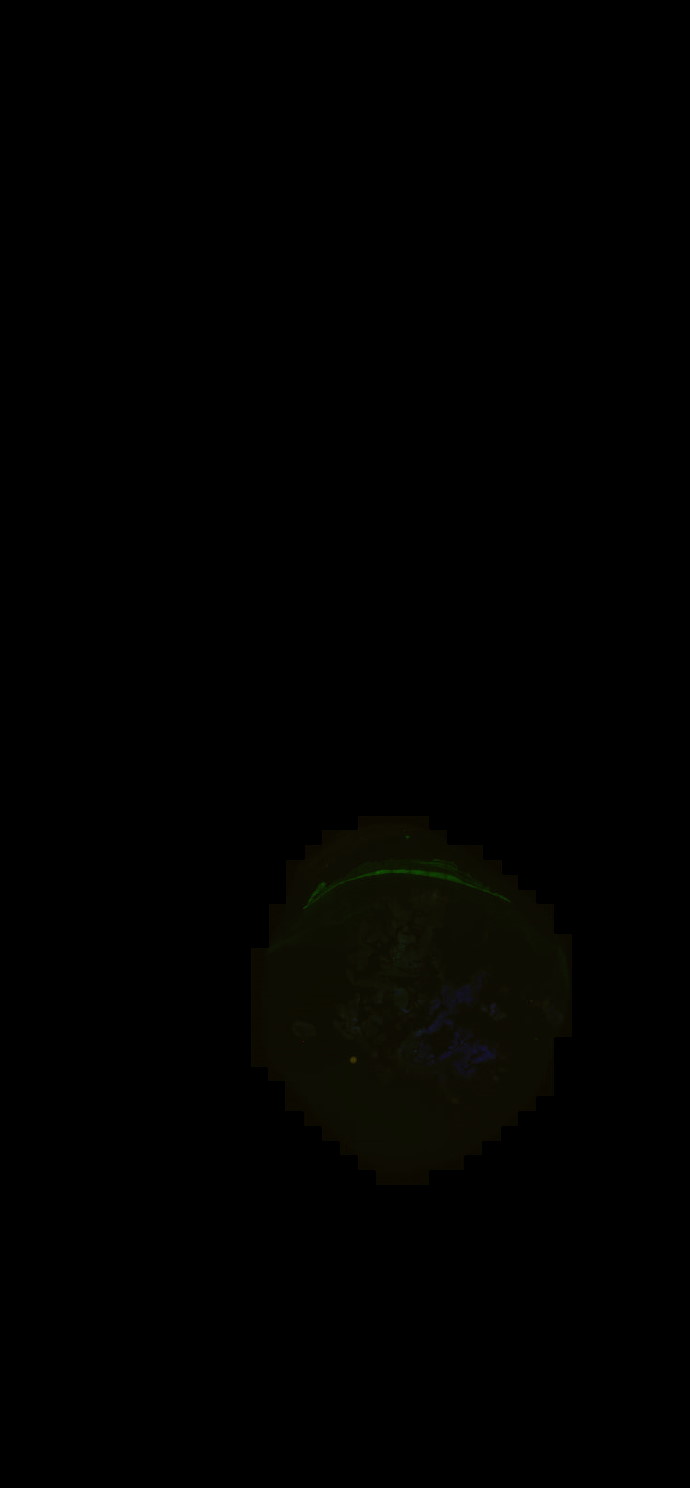

Supplement: Supplementary file 4 — Additional file 4. [file 13048_2022_979_MOESM4_ESM.zip › parafferin section FISH/sections/section 1.mrxs]
