# Supplementary material for: The molecular mechanism of circRHOBTB3 inhibits the proliferation and invasion of epithelial ovarian cancer by serving as the ceRNA of miR-23a-3p
Source: J Ovarian Res. 2022 Jun 1;15:66. doi: 10.1186/s13048-022-00979-1 (PMC9158168; doi:10.1186/s13048-022-00979-1)
Supplement: Supplementary file 2 — Additional file 2. [file 13048_2022_979_MOESM2_ESM.zip › 2.docx]

1.pSI-Check2 vector:


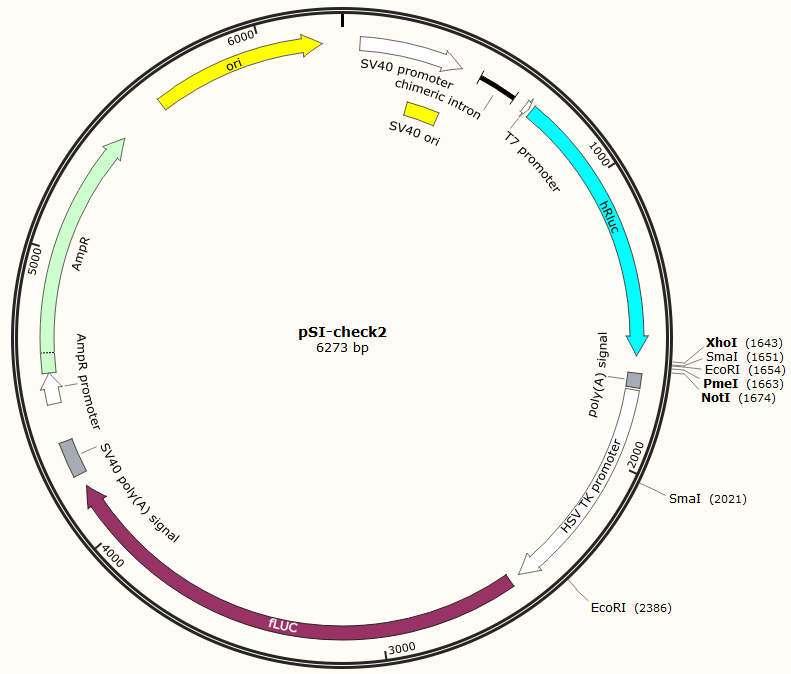


2. seq：

hsa-circ-0007444-wt：
AAAAAATGCCTGTCTTAAAGGCTGAAGCGTCACATTATAACTCTGACTTAAATAACTTGCTGTTCTGCTGCCAGTGTGTGGACGTGGTATTTTACAACCCCAATTTAAAGAAAGTTGTAGAGGCCCACAAGATCGTTCTCTGCGCTGTAAGCCATGTTTTCATGCTGCTTTTCAATGTGAAGAGTCCCACTGACATTCAGGATTCCAGTATCATCCGAACTACCCAGGATCTTTTTGCTATAAACAGAGATACTGCATTTCCAGGTGCTAGCCATGAATCTTCAGGCAACCCACCATTACGAGTCATTGTTAAAGACGCCCTCTTCTGTTCTTGTTTATCAGACATCCTTCGCTTCATTTATTCAGGTGCTTTTCAGTGGGAAGAATTGGAAGAAGATATCAGGAAGAAGTTGAAAGATTCTGGGGATGTTTCAAATGTAATCGAGAAAGTTAAATGCATTTTAAAAACACCAGGAAAG


hsa-circ-0007444-mu：
AAAAAATGCCTGTCTTAAAGGCTGAAGCGTCACATTATAACTCTGACTTAAATAACTTGCTGTTCTGCTGCCAGTGTGTGGACGTGGTATTTTACAACCCCAATTTAAAGAAAGTTGTAGAGGCCCACAAGATCGTTCTCTGCGCTGTAAGCCATGTTTTCATGCTGCTTTTCcAgGaGcAGAGTCCCACTGACATTCAGGATTCCAGTATCATCCGAACTACCCAGGATCTTTTTGCTATAAACAGAGATACTGCATTTCCAGGTGCTAGCCATGAATCTTCAGGCAACCCACCATTACGAGTCATTGTTAAAGACGCCCTCTTCTGTTCTTGTTTATCAGACATCCTTCGCTTCATTTATTCAGGTGCTTTTCAGTGGGAAGAATTGGAAGAAGATATCAGGAAGAAGTTGAAAGATTCTGGGGATGTTTCAAATGTAATCGAGAAAGTTAAATGCATTTTAAAAACACCAGGAAAG


>hsa-miR-23a-3p MIMAT0000078
AUCACAUUGCCAGGGAUUUCC
